# Fuel exploitation and environmental degradation at the Iron Age copper industry of the Timna Valley, southern Israel

**DOI:** 10.1038/s41598-022-18940-z

**Published:** 2022-09-21

**Authors:** Mark Cavanagh, Erez Ben-Yosef, Dafna Langgut

**Affiliations:** 1 Department of Archaeology and Ancient Near Eastern Cultures, Tel Aviv University, 6997801 Tel Aviv, Israel; 2 Laboratory of Archaeobotany and Ancient Environments, Institute of Archaeology & The Steinhardt Museum of Natural History, Tel Aviv University, 6997801 Tel Aviv, Israel

**Keywords:** Environmental impact, Sustainability, Archaeology

## Abstract

Economic and industrial progress frequently comes at the expense of environmental sustainability. For the early Iron Age (~ eleventh to ninth centuries BCE) smelters of the ancient copper industry of the Timna Valley, southern Israel, where today the hyper-arid Aravah Desert provides sparse vegetation, woody fuel for metallurgical furnaces constituted the greatest limiting factor for continued operations. This study presents the first investigation into the fuel sources relied upon by this industry during its most intensive period, as reflected by hundreds of charcoal samples collected from two well-stratified and chronologically anchored accumulations of industrial waste. The two sites demonstrate similar results: a heavy reliance on the local vegetation, particularly *Retama raetam* (white broom) and the ecologically significant *Acacia* spp. (acacia thorn trees), two high-calorific and high-burning taxa best suited for such purposes. It was also observed that over the course of the industry, the search for fuel expanded, as evidenced by the later appearance of taxa unsuited for the prevailing regional conditions, hinting at the detrimental toll the industry took on the local ecosystem. Altogether, it is suggested that the lucrative copper industry ended due to limits in the availability of fuel, caused by anthropogenic hastening of desertification and environmental degradation.

## Introduction

**Figure 1:**
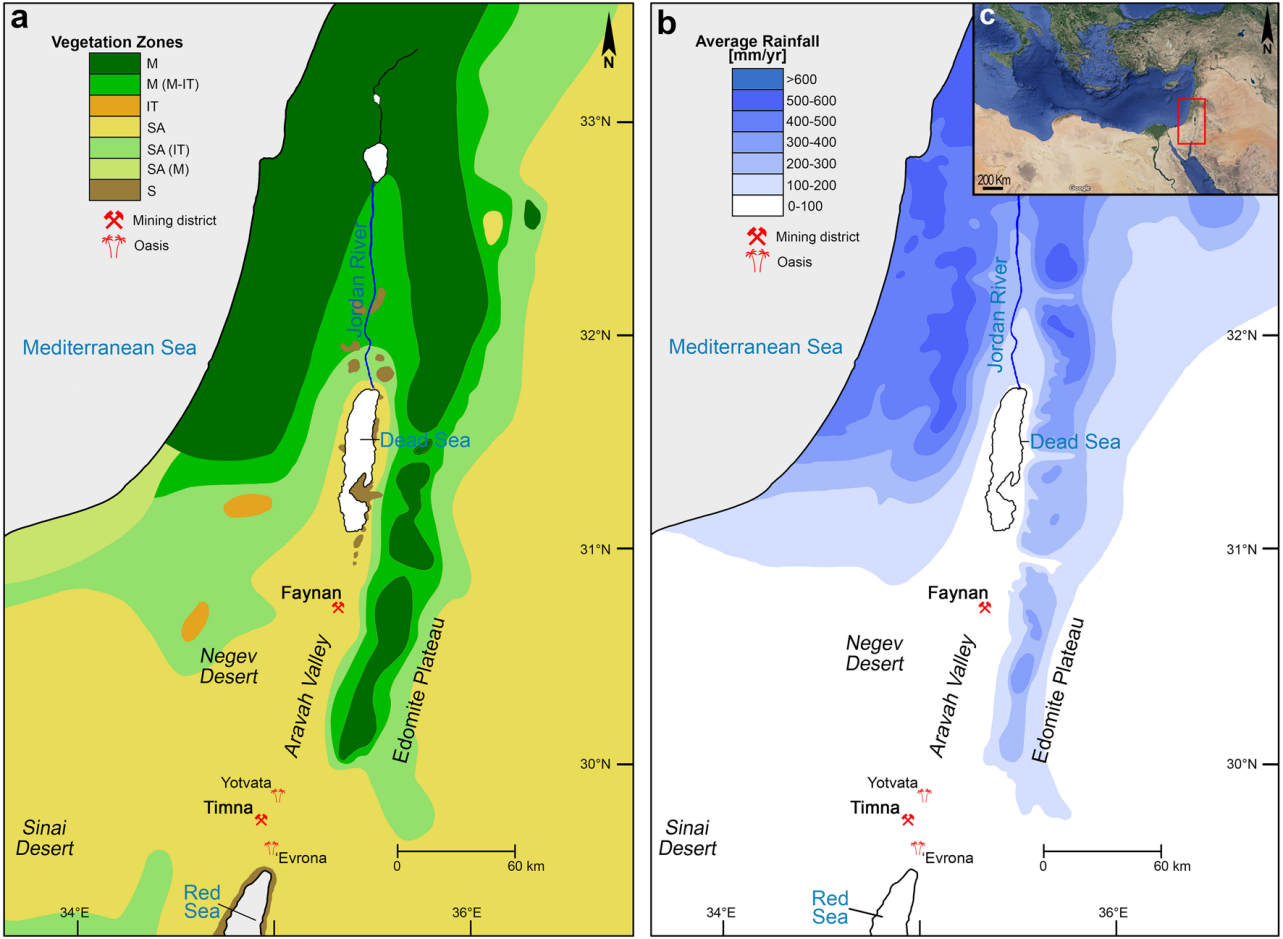
Phytogeographic and rainfall isohyet maps of the southern Levant indicating mentioned sites. (**a**) Distribution of phytogeograpic zones in the southern Levant; M = Mediterranean zone (garrigue, maquis, woodland); IT = Irano-Turanian zone (steppeland); SA = Saharo-Arabian zone (desert); S = Sudanian zone (penetration territory); (**b**) Map of the southern Levant indicating mean annual precipitation in mm; (**c**). Satellite image showing position of southern Levant within the Eastern Mediterranean. Phytogeographic and rainfall isohyet maps created using Adobe Photoshop 2020 ver. 21.1 [www.Adobe.com]. Phytogeographic map created according to^15–18^, rainfall map created according to^19–21^. Satellite image courtesy of Google Earth ( © 2021 Google Earth [earth.google.com]).

**Figure 2:**
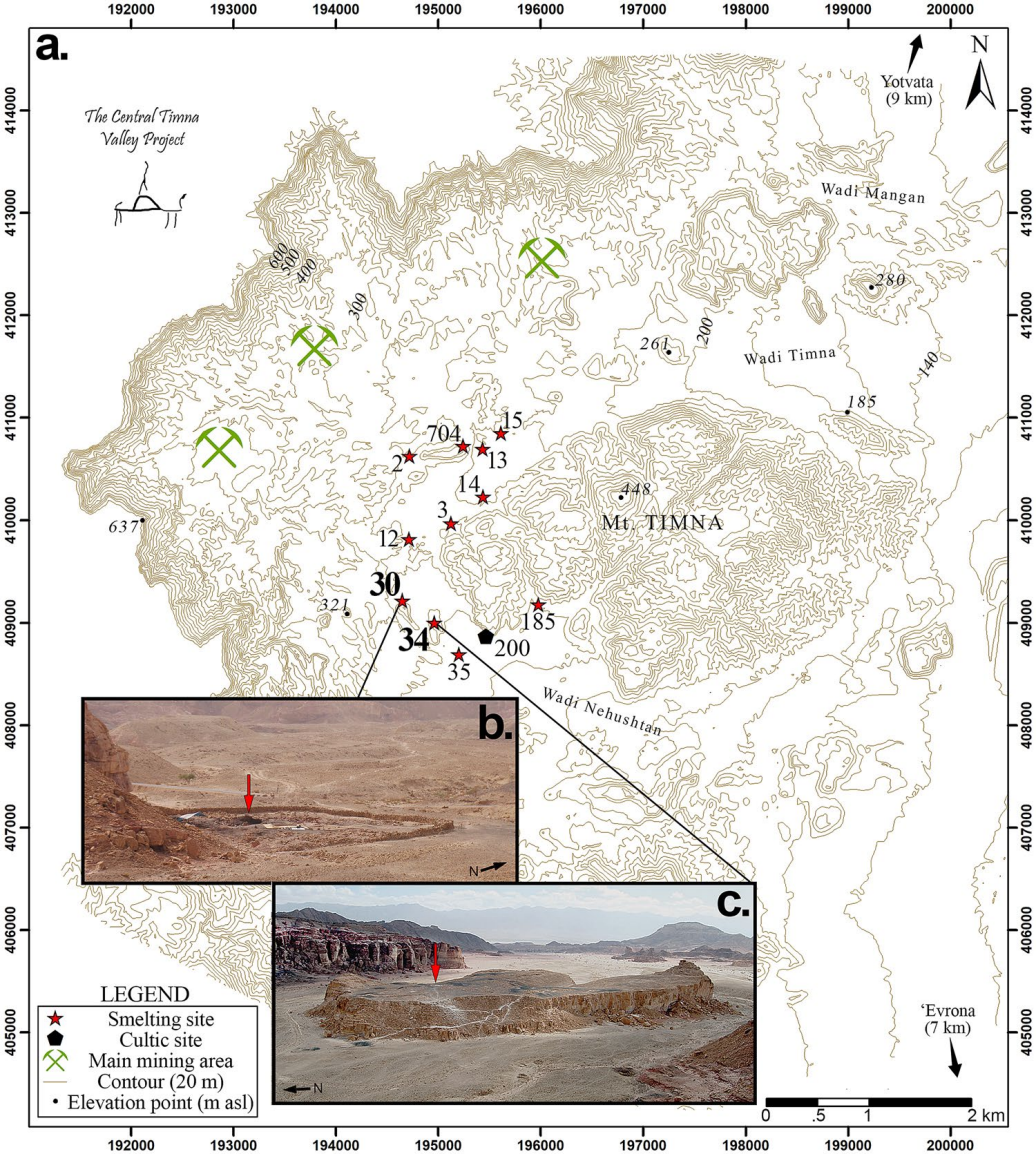
Map of Iron Age Timna Valley and images of Sites 30 and 34. (**a**) Map of the Timna Valley as it appeared in the Iron Age with views of (**b**) smelting site Site 30^5: Fig. 2^ and (**c**). smelting site Site 34/ “Slaves Hill”^6: Fig. 3^. Red arrows indicate location of Slag Mound S at Site 30 and Slag Mound 19 at Site 34. Map generated using ArcGIS for Desktop V.10.4.1 (www.esri.com) and modified using Adobe Photoshop 2020 ver. 21.1 [www.Adobe.com]. Photos taken by E. B-Y.

Along the southern stretch of the hyper-arid Aravah Desert, ca. 20 km north of the Gulf of Eilat, hidden within the sheer cliffs precipitously rising over 500 m to the southern Negev above, lies the Timna Valley (Fig. 1). Since as early as the Chalcolithic (fifth millennium BCE), this extreme environment was host to one of the most significant districts of copper metallurgy in antiquity^1,2^. Though some of the earliest evidence of extractive metallurgy is attested to here, it was during a roughly half-millennium period within the Late Bronze and Iron Ages (thirteenth-ninth centuries BCE) that the Timna Valley achieved its industrial peak^3^. Originally attributed solely to the 19th and 20th Dynasties of New Kingdom Egypt (Seti I-Ramesses V, ca. 1300–1140 BCE) due to the rich corpus of Egyptian finds from a small temple dedicated to Hathor (Site 200^4^), recent excavations and the results of radiometric and archaeomagnetic analyses^5,6^ have revealed that this period of Egyptian hegemony marks only the beginning of the region’s most intensive period of metallurgical activity until the modern era. At the end of the Late Bronze Age, coincident with the collapse of other major and influential polities across the Eastern Mediterranean^7,8^—including the Cypriot copper industry (Alashiya) that had been the main supplier of copper throughout the Mediterranean—Egypt, weakened by internal struggles, withdrew from the region^9^, leaving the lucrative industry in the hands of the local nomadic society (identified with biblical Edom^10^). The ensuing period of ca. 300 years of intense production serves as the backdrop for the research presented here. Following Egypt’s departure, operations continued within numerous scattered unwalled sites throughout the Timna Valley, while the copper mines in the Wadi Faynan copper ore district of the northern Aravah also reopened on a massive scale as part of the reorganization of production in the entire region by the local tribes^11,12^ (Fig. 1). Towards the end of the eleventh century BCE, all smelting activities within the Timna Valley were consolidated within two fortified sites—Site 30 and Site 34/the “Slaves’ Hill” (Fig. 2). During the late eleventh–tenth centuries BCE, a period of increasing social complexity in the southern Levant that witnessed the emergence of the first territorial kingdoms in the region and when the biblical kings David and Solomon are believed to have reigned in the north, the Timna Valley copper industry reached its zenith; several thousand tons of copper slag, still present in large mounds on the surface within the two walled sites, attest to the scale of production that took place during this period^3^. This phase lasted until the latter part of the tenth century BCE, at which point smelting activities ceased at Site 34, being further consolidated within Site 30 alone, and a new smelting technology was introduced^3,13^. This final phase of activity, which started following the campaign of Sheshonk I into the region^3^, ended by the middle of the ninth century BCE, whereupon copper production within the Timna Valley ceased for nearly a thousand years, resuming—on a much-reduced scale—only in the Nabatean/Roman period^14^.

While the exact reason for the halt in metallurgical activities in the Aravah remains unclear—with the campaign of Hazael and the Arameans into Canaan^22–24^ and the reestablishment of Cyprus as a copper production center^25^ usually cited—the discussion tends to focus on Faynan and the northern Aravah, where operations continued on a much larger scale and for half a century longer than at Timna. This research considers the role of the environment in the sustainability of the Timna industry, where woody fuel, necessary for all matter of life (cooking, heating, lighting, and—of particular significance for this industry—its role within the *chaîne opératoire* of copper smelting^26^), was assumed to have been gathered from the immediate vicinity of the sites. Two charcoal assemblages collected from Slag Mound S at Site 30 and Slag Mound 19 at Site 34 reflect the industrial fuel remains from this critical period of activity in Timna, and are able to elucidate the relationship between the ancient smelters and their desert environment, and how such an industry was, or perhaps was not, sustained. Consequently, this study may be able to explain why the lucrative large-scale metallurgical activities ceased.

## Research area

### Geographic setting

The Aravah Valley is a low-lying and geographically restricted desert stretching roughly 165 km from the Dead Sea in the north to the Gulf of Eilat in the south (Fig. 1). It is bordered along its eastern margin by the steep fault escarpments of the Edomite Plateau, while along its west it is flanked by the more gradual slopes of the Negev Highlands^20^. Climatically, the Aravah Valley is the hottest and driest area in the entire Negev/south-western Jordan region and is classified as hyper-arid^20,27^. The mean annual rainfall in the Aravah ranges between 30 mm in the south near the Red Sea, to 50 mm in the north close to the Dead Sea (Fig. 1b). The mean annual temperature in the Aravah is 23–24 °C, though highs of 45 °C can be achieved during the summer, and during winter nights the temperature can drop below zero^28,29^. The arid to hyper-arid climate of the Aravah, as well as the constant threat of droughts, are significant obstacles for societies dwelling in the region, both in the present as in the past^29,30^.

There are few available paleoclimate records for the northern desert regions of the southern Levant, where most paleoenvironmental studies are derived from locations either within or directly influenced by the Mediterranean climate system. The Timna Valley, part of the Saharo-Arabian Desert Belt, is mostly outside of the influence of this climatic system and over 100 km away from the Mediterranean vegetation zone (Fig. 1a). Instead, today, as in the past, the Aravah receives most of its rain through the typically relatively dry Red Sea Trough^31,32^. Though it is therefore not expected that the fluctuations in paleoclimate witnessed in other climate zones will have as marked an effect on the vegetative environment of the Aravah, the valley does nonetheless derive much of its water from runoff from the large drainage area surrounding it^33^. The paleoclimate of the Mediterranean zone of the southern Levant has been reconstructed using multiple proxies, including reconstructions of Dead Sea lake levels^34–36^, detailed pollen studies^37–39^ and isotopic analyses of cave deposits^40–42^. Following a period of increasing humidity that lasted from the Middle Bronze Age II-III (ca. 1750–1550 BCE) to the end of the Late Bronze Age, a sudden and pronounced dry event—the driest period during the Bronze and Iron Ages—occurred during the thirteenth century BCE and lasted roughly a century and a half. The Iron Age began (ca. 1100 BCE) with a brief moist interval^7,38,39,43^ during the Iron Age I and IIA followed by a moderate period through the Iron Age IIB-C^44,45^. It is assumed that the climate regime during the examined period was similar to that of today^45^.

Currently the Aravah Valley is characterized by sparse Saharo-Arabian halophylic and xerophytic vegetation (Fig. 1). In this hyper-arid situation, edaphic conditions and microtopography are the most important factors affecting the moisture regime and the distribution of plant communities^33^. Vegetation develops mostly in a contracted pattern along the wadi channels, as well as in oases^46,47^, such as the ‘Evrona and Yotvata oases, both of which are found ca. 10–15 km from the Timna Valley (Fig. 1a). Species of *Acacia* (thorn trees, including *Acacia raddiana*, *A. tortilis*, and *A. pachyceras*) are the most characteristic trees of this desert environment, and are a keystone species of the ecosystem, as animal and plant species alike rely on them for food, nutrients, and shade^48,49^. Other salt- and heat-tolerant plant species, such as *Calligonum comosum* (fire bush)*, Retama raetam* (white broom), *Zygophyllum dumosum* (bushy bean caper), *Haloxylon persicum* (white saxaul), and *Salsola* spp. (saltworts) are found in the alluvial fans, sandy hills, and chalky marls, though these tend to be shrubs and bushes rather than large trees. Within the oases, in areas with fresh or brackish water and saline soils, *Phoenix dactylifera* (date palm) and *Juncus arabicus* (rushes) grow, while other halophytic species, such as *Nitraria retusa* (nitre bush) and *Suaeda monoica* (seepweed) are present in belts around the salt marshes and oases. Trees of *Tamarix nilotica* and *T. aphylla* (tamarisks) grow along the channel of the Wadi Aravah^33^.

### Site 30 and Slag Mound S

**Figure 3:**
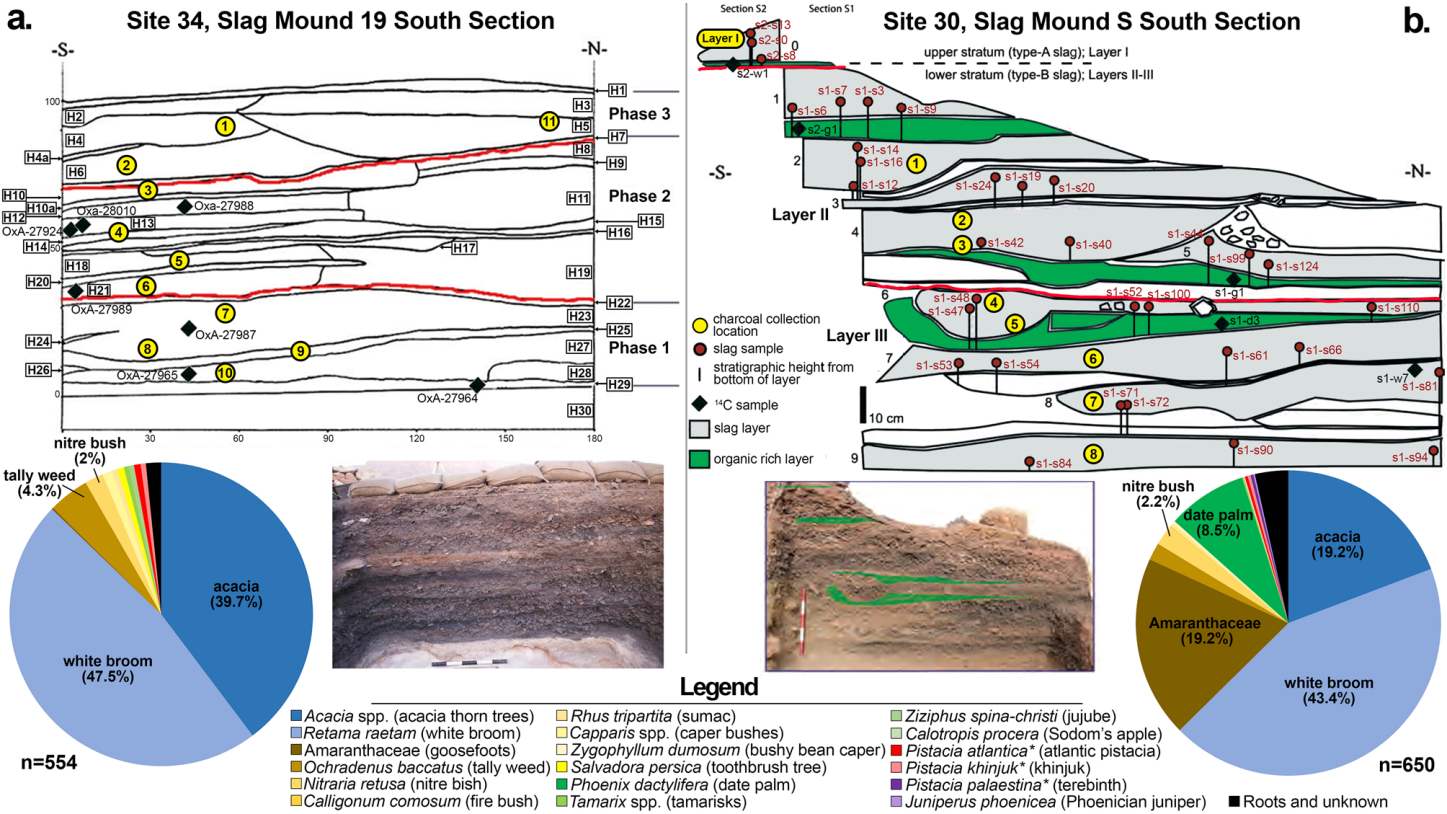
Images and section drawings of sampled slag mounds, (**a**) Slag Mound 19 at Site 34 and (**b**) Slag Mound S at Site 30. Probe locations (yellow circles) as well as radiocarbon sample locations (black diamonds) are indicated with assigned Field IDs. Absolute percentages of identified charcoal from each slag mound are given in pie charts. See Supplementary Tables 1 and 2 for results of radiometric analyses relevant to the dating of both slag mounds. Legend entries with asterisks indicate species-types.

Site 30, a walled smelting camp ca. 4000 m^2^ in area, was first excavated by the Aravah Expedition in 1974 and 1976^13,50^. The excavators identified three “Layers” of activity at the site: Layers III and II, earlier phases similarly characterized by smaller furnaces and iron-fluxed slag, and Layer I, typified by novel metallurgical technologies—such as larger furnaces and tuyères (blowpipe nozzles) and the use of a manganese flux—not found anywhere else in the Timna Valley^50^. In 2009, a section of the large, partially excavated slag mound (Slag Mound S; Fig. 3b) centrally located within Site 30 was sampled for reevaluation^5^. The complex stratigraphy of the mound—an accumulation of slag dumps (i.e., discarded refuse of smelting events also abundant in charcoal) broken by layers of organic-rich material as well as sandy accumulations, possibly reflective of brief periods of abandonment—represents a significant portion of the occupational history of Site 30 (Fig. 3b). The archaeomagnetic intensity of slag and radiometric results of short-lived organic material (such as date and grape seeds) collected from these layers produced a tight chronology lasting from the end of the twelfth century to the ninth century BCE^5^, i.e. from a short period after the Egyptian withdrawal to the end of the Iron Age copper industry, radically shifting the chronological framework away from the (solely) Late Bronze/Egyptian New Kingdom paradigm established by the previous excavators.

### Site 34/ “Slaves’ Hill” and Slag Mound 19

One major focus of the renewed research on the Timna Valley, commenced in 2012 by the Central Timna Valley Project of Tel Aviv University (https://www.tau.ac.il/~ebenyose/CTV/), was the extensive surveying and excavations of Site 34, the largest smelting camp in the valley. Located less than 250 m from Site 30, Site 34 is a low sandstone mesa, ca. 3 ha in area, whose steep cliffs provide limited access to the site. Originally termed the “Slaves’ Hill” by the first surveyors and excavators of the valley due to its restrictive access and fortification walls, which gave the impression of a prison camp^51^, the recent excavations have suggested the opposite: that the workers of the smelting camp were esteemed individuals fed choice cuts of meat and granted access to food and supplies coming from the distant Mediterranean region^52,53^, and were adorned in intricate textiles dyed using innovative and complicated chemical procedures requiring exotic plants and mollusks^54,55^. One large slag mound—Slag Mound 19—was excavated in 2013 (Fig. 3a), and similarly to Slag Mound S at Site 30, provided a complex but comprehensive accumulation of layers (“horizons”) of industrial refuse, organic-rich sediments, and sandy layers indicating brief periods of abandonment that altogether reflect the entirety of Site 34’s occupation. A series of radiocarbon dates taken from short-lived samples collected from the section of Slag Mound 19 revealed that the site was occupied from the late eleventh century into the latter half of the tenth century BCE^6^, i.e. ca. 100 years after the beginning of operations at Site 30 and ending prior to Site 30’s Layer I. Three “Phases” of activity were identified within the slag mound (and also discerned in other areas of excavation) on the basis of the deposition of the slag horizons, which suggest that over the course of the site’s ca. 100 years of activity, the arrangement and positions of the smelting-related activities surrounding the slag mound shifted somewhat, though the activities themselves remained consistent.

## Results (Figs. 4, 5; Supplementary Table S3–S5; Supplementary Figs. S1-S3)

**Figure 4:**
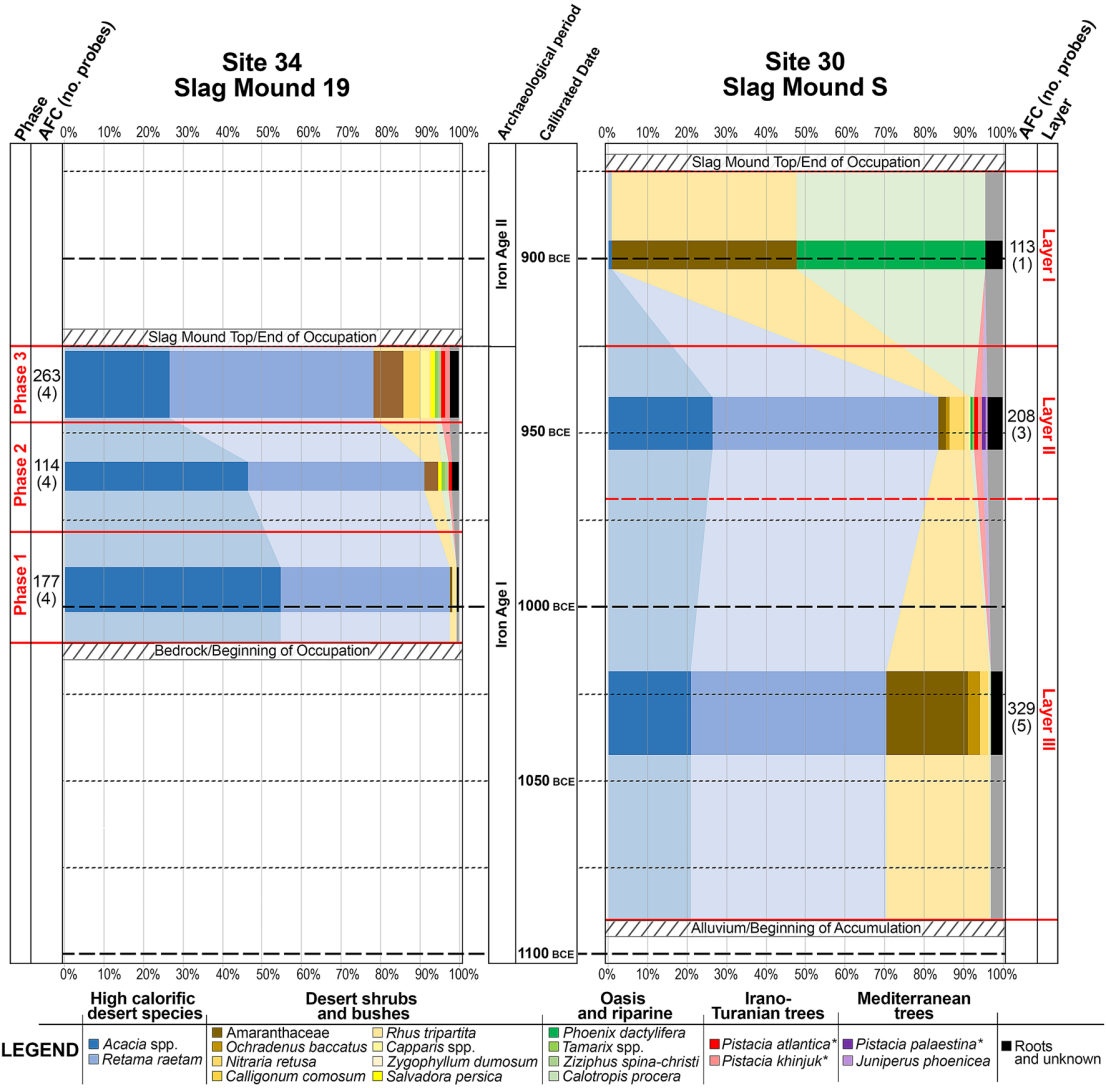
Charcoal spectra reflected within Site 34 and Site 30. Results are given according to percentage values within each sampled Phase/Layer and arranged according to sampled horizon and modeled radiometric dates. Absolute Fragment Counts (AFC) of total examined samples are given along with the number of probes made into each Phase/Horizon given in parentheses. Size of bars are weighted according to the AFC of each Phase/Horizon (see Fig. 3 for slag mound horizons and sampling locations and Supplementary Fig. S1 for results of charcoal spectra according to probes). Legend entries with asterisks indicate species-types.

**Figure 5:**
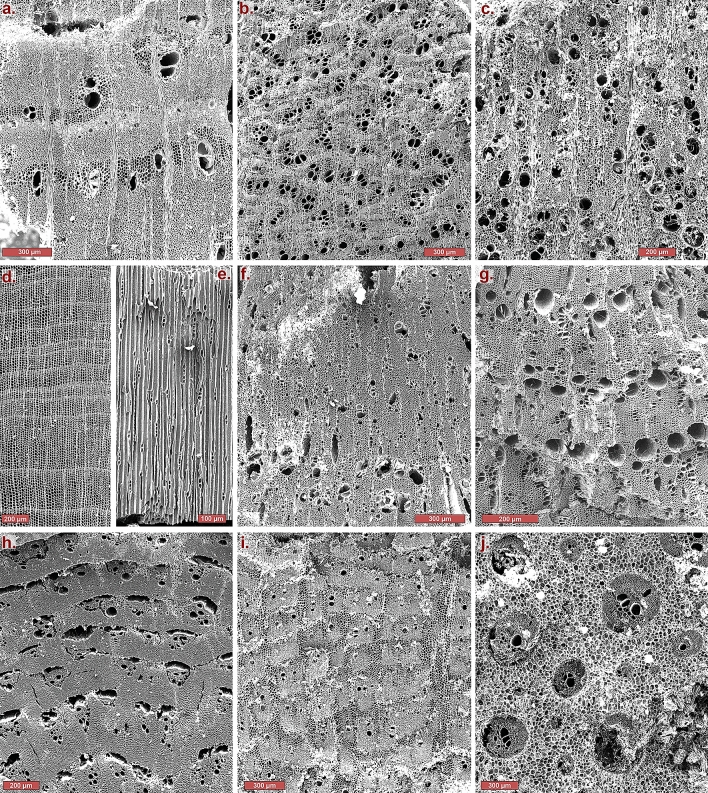
Scanning electron microscope images of select identified taxa with collection locations indicated in parentheses: (a) *Acacia* sp. (Site 30, Probe 1), transverse; scale 300 μm (b) *Retama raetam* (Site 30, Probe 5), transverse, scale 300 μm; (c) *Retama raetam* (root; Site 30, Probe 5), transverse, scale 200 μm; (d) *Juniperus phoenicea* (Site 30, Probe 2), transverse, scale 200 μm; (e) *Juniperus phoenicea* (Site 30, Probe 2), tangential, scale 100 μm; (f) *Pistacia atlantica*-type (Site 34, Probe 3), transverse, scale 300 μm; (g) *Pistacia palaestina*-type (Site 30, Probe 2), transverse, scale 200 μm; (h) *Atriplex halimus*-type (Amaranthacaea; Site 30, Probe 2), scale 200 μm; (i) *Suaeda fruticosa*-type (Amaranthaceae; Site 30, Layer I), trans, scale 300 μm; (j) *Phoenix dactylifera* (Site 30, Layer I), transverse, scale 300 μm. See Supplementary Figs. S2-S3 for additional microscope images.

In total, 1204 individual pieces of charcoal were examined for this study, 554 from Slag Mound 19 of Site 34 and 650 from Slag Mound S of Site 30 (Figs. 3 and 4). By far the most heavily represented taxon was white broom (*Retama raetam*), which comprised 45.3% of all examined pieces, or 43.4% and 47.5% of the Site 30 and 34 assemblages, respectively. Acacia trees, (*Acacia* spp.) constituted the second-largest represented taxon, comprising 28.7% of the combined assemblages or 21% of the Site 30 assemblage and 39.7% of the Site 34 assemblage. These two taxa collectively account for three quarters of all examined samples. In addition, a large array of desert shrubs are reflected in smaller numbers. Amaranthaceae/Chenopodiaceae (goosefoots), a family of annual and perennial shrubs and sub-shrubs well-represented among halophytic and thermophilic flora, comprised the third-largest taxon if considering all examined samples; however, while they comprise 20.7% of the Site 30 assemblage, they are almost totally absent within the Site 34 assemblage (< 1%). Many of the species in this family have similar anatomical structures that make identification to the species or even genus level difficult if not impossible (e.g.^56^); nonetheless, an attempt was made at differentiation within the Site 30 goosefoot assemblage on a comparative basis with modern references (see Materials and Methods below and Supplementary Material S1 for more details on sampling and methodology), and the following species-types were assigned on this basis: saltworts (*Salsola baryosma*-type, 6.2%; *S. vermiculata*-type, 4%; *S. tetrandra*-type, < 1%), Mediterranean saltbush (*Atriplex halimus*-type, 3.7%), *Aellenia lancifolia*-type (< 1%), shrubby seablight (*Suaeda fruticosa*-type, 1.5%), eshnan (*Seidlitzia rosmarinus*-type, < 1%), white saxaul (*Haloxylon persicum*-type, < 1%), and anabasis (*Anabasis setifera*-type*,* < 1%). The wide variety of types witnessed within the goosefoot group suggests that either (as suggested) a variety of different species were used, and/or different parts (branches, stems, trunks) of plants of this group were used (e.g.^57^). Other desert shrubs included tally weed (*Ochradenus baccatus*, 1.9% and 4.3% of the Site 30 and 34 assemblages, respectively), nitre bush (*Nitraria retusa*, 2.2% and 2.1%), fire bush (*Calligonum comosum*, < 1% at Site 30), caper bushes (*Capparis* spp., < 1% and 1.2%), and bushy bean caper (*Zygophyllum dumosum,* < 1% at Site 30). Few taxa that grow in the oases or nearby water sources within the desert were also identified: tamarisks (*Tamarix* spp., < 1% at both sites), Sodom’s apple (*Calotropis procera*, < 1% at Site 30), jujube (*Ziziphus spina-christi*, < 1% at Site 34) and date palm (*Phoenix dactylifera*, 8.5% at Site 30 overall, though 47.8% of the Layer I charcoal). Few species (each comprising < 1% of the assemblages) not presently encountered in the southern Aravah were identified as well: these include sumac (*Rhus tripartita*, found at both sites) and the toothbrush tree (*Salvadora persica*, encountered at Site 34), two desert elements still present in the Dead Sea region and, in the case of the toothbrush tree, along the Edomite Plateau, while sumac is still present in one stand in the southern Negev Desert. Phoenician juniper (*Juniperus phoenicea*), a coastal Mediterranean element with extensions into desert regions, was found at Site 30. *Pistacia* spp., pistacia trees, were also encountered, with three species-types within the *Pistacia* genus identified according to existing and observed precedents (and following the criteria of^58^): Atlantic pistacia (*Pistacia atlantica*) and khinjuk (*P. khinjuk*), Irano-Turanian relict trees identified at both Site 30 and Site 34, and the terebinth (*P. palaestina*), a Mediterranean element identified at Site 30. Roots of certain taxa—in particular white broom—were also identified, as were unknown types (many likely also deriving from roots), the latter amounting to 2.8% of the total assemblage, or 3.5% and 1.6% of the Site 30 and Site 34 assemblages. In addition, evidence of fungal hyphae were also noted in some samples (see Supplementary Fig. S2: f and m), raising the possibility that dead wood was also at times collected (a less labor-intensive task than harvesting from a living tree, with the added benefit of having much of the original moisture content already removed); however, as fungi may attack living trees as well, it cannot be ruled out that living wood infested with fungi was also gathered^59^.

## Discussion

Given the context of the present assemblage within well-stratified and chronologically anchored slag mounds located in a marginal, arid and geographically restricted environment, many of the variables that may typically induce interpretative and taphonomic biases within an anthracological study (e.g.^60–62^) are able to be accounted for or lessened. Charcoals extracted from the slag mound reflect human choices, as opposed to natural accumulations; their association with metallurgical activities (versus domestic ones such as heating or cooking) is clear from the other archaeological signatures of the site and contents of not only the slag mounds but particularly those horizons probed within the slag mounds (e.g. the high levels of crushed slag and fragments of smelting furnaces). While it is possible that other activities are reflected within the assemblages, their contribution is likely minute in comparison to that of the industrial activities (though a differentiation between different stages within the smelting process, such as primary or secondary smelting, cannot be soundly performed). Moreover, while reconstructions of the smelting furnaces are near impossible to reproduce, as they were destroyed following every smelting event in order to extract the copper produced, smelting was a relatively standardized process, at the end of which the remaining charcoal and metallurgical debris were dumped onto the slag mounds, creating, over time, successive layers of smelting events that sealed and secured earlier events for the archaeological record. Since their deposition, the dry environment and isolated nature of the site has likewise aided in lasting preservation. High fragmentation was most prevalent among the goosefoot members, as their secondary phloem creates natural weak points and cleavage planes, but the examination of samples as small as 5 mm was employed in order to compensate in part for this potential bias in results. The two assemblages, observed both in the higher resolution of the individual probes and the lower resolution of the site-wide phases or layers, allow us to interpret the results largely as an “average” of the industrial fuel preferences used over time.

Most woody taxa represented within the two assemblages largely reflect the vegetation present in the southern Aravah today. This is consistent with Site Catchment Analysis (SCA^63^) and the Principle of Least Effort (PLE), according to which resources are gathered from the immediate vicinity of the settlement or site, i.e. what is easiest and hence requiring the least effort to procure^60,64–67^. This means that, unlike certain foods and other goods that had been brought from afar to sustain the mining communities^53^, fuels were not being subsidized through long-distance trade (despite the significance of the copper trade itself). This further suggests that during the Iron Age, the vegetation of the southern Aravah consisted of a variety of desert and savannoid flora similar to what is witnessed today (Fig. 1;^19,47,68^): acacia trees along with desert shrubs in the steppe zones and wadi beds, desert scrub and shrubs in the dune sands and reg, small halophytes (e.g. the salt tree and Mediterranean saltwort) in the salinas (salt marshes), while closer to oases other varieties of larger trees (including tamarisks, jujube, and Sodom’s apple) and date palms grew. A similar phenomenon was observed in the charcoal studies conducted at the Wadi Faynan copper smelting sites, where it was generally observed that the ancient smelters gathered their fuels from the immediate vicinity of the smelting sites^69–74^, though, unlike Timna, Faynan benefits from a greater abundance of water and vegetation due to its location at the base of the Edomite Plateau (Fig. 1). Somewhat conversely to Timna, however, acacia trees are near absent within the Faynan charcoal assemblages (in both domestic and industrial contexts), even though they are abundant in the area, while the white broom maintains a high frequency of use here. This may in fact be evidence for a cultural preference *against* the use of acacia trees, should other options be available^75^, a phenomenon observed among the Bedouin and other nomadic groups in the modern era (see also below)^76–80^.

While on the one hand, the PLE suggests that resources are gathered from nearby the site, on the other hand, it also supposes that resources are gathered in direct proportion to their presence in the environment. What is witnessed within the slag mounds, however, is not this case. Instead, two taxa dominate the Site 30 and 34 assemblages—acacia trees and white broom bushes. At Site 34, where Slag Mound 19 reflects roughly a century (ca. 1010–920 BCE; Supplementary Table 2) of dedicated and intensive smelting activities, these two species together make up nearly 90% of the entire corpus of examined samples. At Site 30, reflecting slightly more than two centuries of activity (ca. 1100–870 BCE; Supplementary Table 1), the overall distribution is somewhat more shared between the preferred species of acacia and white broom (collectively making up roughly 63% of identified samples, see Fig. 3b) and the other varieties of local desert shrubs. However, if only the Layer II-III samples (ca. 1100–920 BCE) are considered—i.e. from the period prior to the technological and fuel preference changes of Layer I (ca. 920–870 BCE)—the percentile of acacia and white broom jumps to nearly three quarters of utilized fuels (Figs. 3 and 4). Though a great deal of the biomass present in the desert regions is provided by acacia, the largest and most abundant trees growing within the desert ecosystem, their size alone cannot account for the relatively low representation of other species in the slag mounds. Similarly, the dominance of white broom over both acacia as well as other bushes found in the region requires some explanation. The white broom is a medium-sized bush usually growing to heights of 1–3 m, though they also have thick, poorly branching and mainly vertical roots able to grow up to 20 m long, and exposed taproots that can grow up to 2 m long^81^^:47–48;^^82^^:74^. These two taxa, bearing particularly thick fibers, have high wood densities and therefore high calorific values^73^, physical–chemical properties that make them hotter-burning fuels than most of the other desert bushes (e.g.^83^; particularly in the case of the rather abundant goosefoot species, which also tend to fragment as charcoal due to their anatomical structures). The hot-burning charcoal of the white broom is even referenced in the Hebrew Bible (Psalms 120:4), while the roots of this bush are also acknowledged (Job 30:4). It is therefore suggested that the ancient smelters were familiar with and understood the effects of the properties of these particular taxa, causing them to be preferential for smelting over the other taxa, which instead would have been used for other activities taking place within the larger mining community likewise requiring woody fuel, such as cooking, heating, lighting, ceramic production, etc. Evidence for such behavioral preferences has been observed since as early as the Mesolithic, such as at the site of Sibudu (77–65 ka YBP) in the KwaZulu-Natal region of South Africa, where the ancient inhabitants seem to have already been aware of the varieties of wood in the area and their different properties (particularly their burning temperatures vis-à-vis their wood densities), and selected their wood accordingly^84^, though such selective preferences may have begun even as early as the Paleolithic^85^. This may also explain the change in goosefoot representation within the Site 30 assemblage: while in Layer III, goosefoots make up 20% of the overall assemblage and are well-represented in every probe from this layer, in Layer II, their representation shrinks substantially to less than 2%. While Layer III begins prior to the activities at Site 34, once Site 34 becomes active—a period largely contemporaneous with Layer II of Site 30—the two assemblages more closely resemble each other. It is possible that the higher representation of these “lesser quality” taxa in the Site 30 assemblage is due to the site being much smaller and compacted than the more expansive Site 34, with activities besides smelting taking place in the immediate vicinity of Slag Mound S (as attested to by the ceramic kiln found adjacent to Slag Mound S^50^), while it is also possible that these taxa were used in the initial charges at the start of the smelting process. However, the near absence of this family in Site 34 (established as a dedicated and protected smelting camp) and their diminished representation in Layer II of Site 30 may reflect a shift in resource management beginning in the late eleventh century, in which the white broom and acacia (along with some of the heartier and more densely fibered small bushes such as tally weed and nitre bush, the latter of which is still used as a fuel source among Bedouin communities^81^) were specifically selected for smelting purposes. Meanwhile, the goosefoots may have been earmarked for other activities (cooking, heating, lighting) or even saved for the grazing flocks that accompanied the metalworking communities.

Of the desert elements identified within the assemblage, of particular note is the toothbrush tree, a Sudanian relict species characteristic of savannahs in the dry tropical regions of Africa^81^,^86^^: 32^. It is not present in the southern Aravah today, though it is still present in enclaves in the southern Levant where the microclimate supplies enough water^18,33,86^, primarily around the Dead Sea where springs are more abundant. It seems apparent that a greater array of desert and savannoid vegetation had been available within or around the Timna Valley certainly at the start of the Iron Age than is seen today. It is reasonable to infer that during the Iron Age water was more abundant within the local microclimate, contained in great part within the plants and soils and maintained in the water cycle of the ecosystem through evapotranspiration, but the removal of these vegetative elements irreversibly affected the system's ability to retain moisture (e.g.^87^). Many of the plant taxa in semi-arid and arid environments are tolerant of stresses related to salt, heat, and brief periods of drought, and the continued presence of the toothbrush tree into the Iron Age may be the product of plant resilience in a relatively stable ecological situation, i.e. one without the added pressures of human industrialisation.

The sequence of events witnessed in Site 34 in particular, and bolstered by the results of Site 30, suggests that over time the smelters needed to expand their culling range, up the wadis and to the oases, in order to gather fuels. While at its onset the smelters of Site 34 relied almost exclusively on acacia and white broom, these taxa decrease over time replaced in part by flora typically requiring a greater degree of precipitation than is afforded within the Aravah itself. The ratio of acacia steadily decreases in Site 34 over time, replaced instead with white broom and other taxa. White broom maintains a relatively high percentage in most horizons along the sequence, though it shrinks in its representation in the latest probed horizon (*34–1*) where it makes up only about one third of examined samples, at which point it is subsidized by other taxa as well (Figs. 3 and 4, Supplementary Fig. S1). In this final horizon, these “less-preferred” taxa make more than 30% of the identified taxa, while unknown varieties (likely younger samples with underdeveloped anatomies) comprise 7%. This accords with the final assumption of the PLE, which suggests that resources would be gathered from further afield only when the nearby resources become depleted^65^. While some fuel resources may have been provided by floods, which can carry wood long distances, the catchment area feeding water into the Timna Valley is relatively small. The presence of fungal hyphae in some samples may attest to this phenomenon, though this is just as likely the reflection of opportunistic harvesting of in situ dead and dry wood, or perhaps even the collecting of infested living wood.

Irano-Turanian and Mediterranean trees which today are found only in areas of higher elevation and precipitation begin to appear at a similar time in both sites—around the middle of the tenth century and following. Today, Phoenician juniper is totally absent among the Israeli flora; the closest modern stands to Timna can be found in the northern Sinai and along the Edomite Plateau (Fig. 1;^33^). In the past, however, juniper was a more prevalent element in the region, and though populations were once found in the Central Negev, where vegetation was denser, the most recent evidence of them in the Negev is dated to ca. 8000 cal. BC^88^. The disappearance of juniper in the region since the Early Holocene is most likely due to human pressure on the natural environment, further evidence of which can be found at Faynan. During the Early Bronze Age II-III (ca. 2900–2350 BCE), juniper was a major source of copper smelting fuel at Faynan, but is near absent in the Iron Age assemblages (e.g.^69^). This suggests that the stands presently upon the top of the Plateau may have once extended further down the slopes, and their populations may have been severely reduced by the Early Bronze Age activities coupled with the climate changes that have occurred since the Early Bronze. All three *Pistacia*-types are found in the vicinity of the Aravah Valley and, like the juniper, may have been better represented in the past, particularly along ravines and upon higher elevations where more water was available. Khinjuk, an Irano-Turanian tree, like the juniper, is totally absent from the Israeli flora, and in antiquity as well as today is found in the Sinai (including at the Iron Age II site of Kuntillet ‘Ajrud in the Eastern Sinai^89^) and along the Edomite Plateau^33,90^. The Atlantic pistacia, an Irano-Turanian element, is still found along the Edomite Plateau as well as throughout the Central Negev Highlands^33^. It is totally absent in the Aravah Valley and appears only in one stand in the southern Negev ca. 15 km north of Timna. The closest stands of terebinth, a characteristic tree of the Mediterranean zone, are on the higher elevations of the Edomite Plateau, with their furthest southward extensions found ca. 50 km south of Faynan. This is the only location where all four of the aforementioned species (Phoenician juniper, terebinth, khinjuk, and Atlantic pistacia) are still found together. In the Iron Age, were any of these species still found in or around the immediate vicinity of the Timna Valley, they were likely already relict species represented by few individuals rather than entire stands or large populations.

With the exception of a few ascents, the journey into or out of the Aravah Valley from either the Negev Highlands to the west or the Edomite Plateau to the east is treacherous if not impossible. The Negev hills bounding the western margins of the Aravah rise ca. 500 m above the Aravah Valley; to find even a moderate ascent, one must leave the Timna Valley, while most major routes required travelling to the shores of the Red Sea—or at least Yotvata—before heading westward^91^. In the case of the Edomite Plateau, the cliffs rise to a precipitous height of ca. 1500 m asl. Though the enhanced elevation allows for the average precipitation to achieve ca. 350 mm per annum in some locations^69^—thus fostering a much greater array of vegetation—access to these locations is permitted by even fewer ascents than those entering into the Negev. Moreover, it is assumed that charcoal was produced specifically for the sake of metalworking in the vicinity of the smelting sites, rather than being transported as such. Though charcoal production locations are difficult to identify in the archaeological record and have not been found at Timna, charcoal burns hotter and retains its heat longer than fresh wood, and is also better for the chemical process (due to the removal of water and other volatiles) required for the separation of the metal from the parent mineral^92^, while ethnographic evidence further points to its preference as a smelting fuel among traditional societies (e.g.^93–95^). However, if charcoal is borne over long distances, there is a great risk of the fragile material being crushed into fines (charcoal dust) that would stunt airflow within the smelting furnace, rendering it unsuitable for such a volatile dynamic system^70^. For this reason it is most likely that charcoal was produced nearby the smelting camps, while the wood was culled and transported to the charcoal production centers by groups of people dedicated to the task. For the non-local taxa, a great deal of effort would need to be invested in bringing them from distances of up to 100 km away across steep terrain, likely still as uncarbonized wood.

The geographic isolation of the Aravah made fuel procurement for the Timna copper mines a largely local endeavor. The sudden intensification in the production of copper beginning in the middle of the twelfth century BCE and peaking within the eleventh–tenth centuries BCE, as typified by Sites 30 and 34^3^, therefore would have put a large strain on the local environment. However, unlike the Faynan copper district in the northern Aravah, whose Iron Age industry was fueled largely by fast-growing tamarisks watered by the high water table and perennial streams of the region^69^, in Timna, vegetation would have been more scarce. Consequently, with no signs of stress in ore qualities from the earliest understood stages of copper production in the Chalcolithic on through the Early Islamic period^14,96^, fuel sources, coupled with the scarce availability of water, were a major limiting factor in the ongoing activities in the southern Aravah^97^. Rough calculations relying on experimental data (e.g. ^26,98^) suggest that 10 tons of wood (by weight) were required for the production of one ton of copper^97^^,^^99^^:936–938^, though estimations as high as 80 tons of wood for one ton of copper have also been suggested^98^^:53,147^. It was estimated that 30,000 tons of wood were required for the Iron Age Timna industry^99^^:938^, while the scale of wood used in Site 34 alone is estimated to be at least 3300 tons (if a low estimate of only 500 tons of slag are assumed for Site 34) for a period of about 100 years or somewhat less. Considering the ratios of acacia and white broom present within Slag Mound 19, and taking mean values for the biomass estimates of these plants (8.5 kg for white broom and 325 kg for acacia^73^), this would equate to at least 4100 acacia trees and over 185,000 white broom bushes for this single site.

No taxa are better able to reflect the potential impact of such an environmental strain than the acacia trees, a keystone species within the desert ecosystem. Many forms of life, plant and otherwise, are dependent upon these species for their own survival^49,100^. Species diversity increases as the number of individual trees increases while, conversely, the opposite is also true^101,102^: as the numbers of acacia go down, the number of plant taxa reliant on them will also decrease. This in turn will affect other aspects of the ecosystem, including soil quality and the diversity of faunal species that depend on the acacia and reliant species for forage, shade, and shelter^49^. Acacia are long-lived trees with infrequent periods of recruitment^48,103–105^, with many issues of decreased recruitment in modern contexts especially driven by anthropogenic actions^106–109^. Acacias have also been shown to be susceptible to changes in environmental and ecological conditions, and though in a natural situation the ability for mature specimens to recover following extensive droughts has been demonstrated (e.g.^109^), the acacia population in Israel has decreased by a third over the last several decades on account of unprecedented exploitation of underground water^48,110,111^.

For some modern traditional nomadic societies living in the Middle East and North Africa, such as certain groups among the Bedouin and the Beja, acacias are regarded with reverence and frequently protected and carefully managed^79,112^. Charcoaling is a major source of economic income for these groups, but because the delicate nature shared between the acacia trees and their ecosystems is recognized, the preservation of these trees for future generations is linked with their own preservation. Among the Kushmaan Bedouin clan of the Eastern Desert in Egypt, there is even a belief that ancient forests once grew throughout their desert landscape, but that they were destroyed (interestingly enough) by the overexploitation of acacia trees for the production of charcoal for metallurgical furnaces^112^^: 99^. In their own words, “[c]harcoaling is prohibited among us because without the trees, there are no animals, and no Arabs”^112^^: 109^. The Handandawa Beja clan of the Red Sea Hills have a similar understanding of the acacia’s role in upholding the life cycle: “[w]hen the last tree is gone, it is the end of the world”^79^^: 41^. Nonetheless, in some cases from recent history, the overexploitation of these trees for the sake of producing charcoal has been recorded; Stanley, for example, recounts how in the early nineteenth century, the vegetation in the Sinai had virtually disappeared because of the production of charcoal from the acacias, a situation likely further exacerbated by Muhammad Ali Pasha of Egypt when, in 1823, he compelled certain Bedouin to pay tribute *in charcoal* for an assault they perpetrated on the Mecca Caravan^113^^: 25^.

The special attitude towards acacia witnessed among contemporary autochthonous nomadic tribes of the desert regions is probably reflected in the minimal use of acacia charcoal in early Iron Age Faynan. Though the contemporaneous copper production centers of Faynan and Timna were operated by the same emerging tribal kingdom^3^ (identified with biblical Edom^75^), the difference in the charcoal assemblages between Faynan and Timna lies in the availability of other fuel sources in the former, in particular fast-regenerating hydrophilic plants such as tamarisk^97^, while in the southern Aravah there were no alternatives.

Also of note in the Timna charcoal assemblage is the selection of roots for fuelwood. Comparison with reference samples collected from the research area suggests that roots were relied upon to some degree. Though stem and root wood bear similarities in their morphologies (allowing for positive taxa identifications in some cases for encountered roots), variations nonetheless occur to some degree, primarily with regards to the appearance of pith and the size, shape and arrangement of vessels, rays and other cells^114^. While absolute quantification of roots versus stem and branch wood is difficult, their presence within the assemblages was nonetheless noted, pointing to the high demand for woody fuel in order to sustain the Timna copper industry. Though archaeological parallels for the use of roots are rare, one example can be found from Vallon du Fou in the south of France. Here, the large roots of heather—particularly tree heather (*Erica arborea*)—a small tree or shrub with very dense wood, were extensively dug up and used to produce charcoal since as early as the European Iron Age (eighth century BCE) through the Middle Ages (thirteenth century CE)^115^. Given the large amount of biomass provided by the root systems of white broom in particular, as well as the large representation of white broom within the assemblages, it is probable that a sizeable portion of the fuel assemblage in the Iron Age smelting sites of Timna derived from this part of the plant. Moreover, even if roots are left out of the equation, repeated cuttings of plants above the root line, such as in the case of excessive cropping, will severely reduce a plants vitality and ability to produce fresh growth^116^^:171^. Pruning for the sake of sustainability, as has been suggested at Faynan^73,74^, may have been an option for the relatively fast-growing white broom; however, in the case of acacia, the slow growing nature of this taxon makes this an unlikely scenario. In the case of most members of the Amaranthaceae family, pruning or coppicing would likewise not be an advantageous means of wood sourcing for industrial charcoal production, as many of their branches do not achieve a tremendous volume on their own and the tedium of selecting those branches would produce very little payoff. In this case, roots would be preferred; however, experiments conducted on *Haloxylon salicornicum* (the Rimth saltbush, a small bush within the Amaranthaceae family) suggested that uprooted (= cut ca. 10–20 cm below surface, typically the thickest part of the stem of this species) plants reproduce less than half the amount of fresh biomass compared to surface-cut plants after seven months^117^, highlighting the unsustainability of such a practice. It is also important to bear in mind that it takes several years for a tree (particularly acacia) to produce branches with dimensions suitable for fuel use, and excessive cropping will severely reduce the tree’s vitality and ability to produce fresh growth^116^^: 171^. Of further consideration is the fact that many of the species relied upon by the Iron Age smelters, including acacia and white broom, are also responsible for soil stability, plant recruitment, and halting land degradation^19,33,82,118^ and are significant sources of food and fodder for wild and shepherded animals^119^. Moreover, a great degree of water in arid environments is stored in the roots and stems of the desert vegetation, and by removing them, water is likewise removed from this system, further enhancing desertification rates^87,120,121^. It is also worth noting that in the numerous excursions and field surveys conducted by the authors over the past decade, only two small white broom bushes were encountered in the northern part of the Timna Valley, with no presence elsewhere within the area, raising the possibility that the white broom population has never recovered from the Iron Age overexploitation.

The exploitation of keystone species coupled with the potential uprooting of other available vegetation likely had long-lasting and dire effects on the local ecosystem in and around the Timna Valley. Near the end of the tenth century BCE, metallurgical operations were further centralized when Site 34 ceased activities and smelting took place only at Site 30^3^. This final period, which lasted into the early ninth century, is also characterized by a radical change in the fuels used: instead of acacia and white broom, the assemblage of the uppermost horizon (Layer I) is dominated by date palms (47.8%) and small bushes (46.9%). Today the nearest date palm stands can be found around the oases of ‘Evrona and Yotvata, both ca. 10–15 km away from the examined sites (Fig. 1). While on the one hand, the improved smelting technologies may have allowed for a greater array of plant species to be utilized over those with higher calorific values, on the other hand, the larger furnaces would have needed more fuel generally, and the sudden selection of plants that are not found in the immediate vicinity of the smelting sites (i.e. the date palm) and smaller bushes is probably indicative of a severely reduced environment, where the once preferred fuel sources had been removed, and only the faster growing yet smaller local elements remained. During the Iron Age, date palms were certainly relied upon for their fruits, as numerous date seeds were found during recent excavations^6,53^. The pruned portions of the plants, which were probably cultivated within the oases, may have been selected for fuel, as was suggested for the Iron Age sites in Faynan^74^; however, at Faynan, with its greater access and proximity to water and vegetation, such agricultural practices would have been possible much closer to the smelting sites than at Timna. It is also worth raising the possibility that the palms, which are often used as support beams and roofs for tents when other sources of straight beams are unavailable^122^, derived from the abandoned smelting and mining camps that would have been found throughout the valley when only Site 30 was active. Following this short-lived final stage of the Timna copper industry witnessed only at Site 30, metalworking in the Timna Valley would cease entirely for nearly a millennium.

## Conclusions

Near the end of the eleventh century BCE, the copper industry of the Timna Valley would enter its most intensive period of activity in antiquity. At this time, smelting activities, once dispersed throughout the valley, were consolidated within two newly walled sites—Sites 30 and 34—where thousands of tons of slag^96,98^ still present on the surface of the sites bear witness to the scale of activity that once took place there. In order to produce this amount of slag (itself reflective of the production of hundreds of tons of copper), a commensurately greater magnitude of wood would have been required to fuel the smelting furnaces. With the evidence provided by the charcoal collected from within two slag mounds excavated at these sites, it can now be understood that the wood for these purposes was sourced locally from within and around the southern Aravah Desert itself. Moreover, it is clear that the ancient smelters during this period were aware of the best woody fuels available to them in this otherwise scarce environment, as made evident by the high representation of white broom and acacia trees, two taxa producing dense and high-burning wood. The reliance on these species in particular, however, as well as the use of roots of these and other taxa, were likely to have had a detrimental effect on the delicate ecosystem of this extreme environment, potentially already exacerbated by the climate crisis that occurred at the end of the Late Bronze Age just prior to the period under study. The region would have quickly approached its carrying capacity with the (likely seasonal) arrival of the mining community and their herds, whose sudden and intensive activities would have upset the equilibrium of the ecosystem. The overexploitation of the keystone acacia species, upon which much other plant and animal life depend, would have a ripple effect throughout the entire ecosystem, and together with the uprooting of other desert bushes, would significantly reduce the reproduction and recruitment of the desert flora, limit the available shade and fodder for desert fauna, decrease soil quality, remove much needed water from the system, and ultimately lead to an environmental collapse certainly within the Timna Valley microclimate if not beyond. Evidence for such destruction is implied by the later appearance of taxa (particularly juniper and pistacia trees) unsuited to the extreme conditions of the southern Aravah Desert, suggesting an increasing range of wood collection over time. By the late 10th-early ninth century BCE, a novel and more efficient smelting technology utilizing larger furnaces and requiring even more fuel was in use. In this final stage of the Iron Age industry, when smelting activities were further restricted to Site 30 only, the fuels selected for the furnaces also drastically change; choice fuels all but disappear and small desert bushes and palm trees become the main fuels used. Given that for much of the period examined (especially when Site 34 was active), the selection of fuels had been based on what was preferred first and available second, the change in the selection suggests that what was most readily available—i.e., small bushes and palms that were likely to have grown in abundance only ca. 10–15 km away in the oases (assuming they were not simply the remains of abandoned tents from within the valley itself)—was significantly limited contra what was available less than two centuries prior. The short-lived nature of this final stage of activity, coupled with the total halt of the lucrative copper industry in the Timna Valley for nearly a thousand years, suggests that the diminishing availability of fuel sources in the southern Aravah was a primary reason for the cessation of this industry that had been operating continuously for about four centuries prior.

## Materials and methods

The examined charcoal assemblages derived from two series of dedicated probes made into the western sections of Slag Mound 19 at Site 34 and Slag Mound S at Site 30 in 2014 and 2015. The use of these sections was significant as they both served as the basis for the chronological reconstruction for their respective sites^5,6^, therefore allowing the probes to be correlated both within the established absolute chronologies as well as the relative horizontal stratigraphy for the duration of each slag mound’s use. Probes were made into horizons rich in both charcoal and slag (understood to reflect periods of intense industrial activity) within the slag mounds and create a relative stratigraphy able to reflect changes within each mound’s respective occupational history. Charcoal samples were collected along with the surrounding sediments and materials, including large amounts of crushed slag (some of which still contained fragments of charcoal within). Unfortunately, deterioration of Slag Mound S since its original excavation has resulted in the destruction of the upper portion of the section, preventing sampling from certain contexts. In addition, all of the remaining Layer I material from Slag Mound S (not along the section), including all of the charcoal found therein, was excavated at the time of probing due to its rapidly deteriorating state. An initial count of 500 examined charcoal samples per slag mound was sought for analysis, though this number was increased slightly to increase the resolution of certain probes and/or Layers/Phases. For Site 30, this number was further increased due to both the greater duration of time reflected within this slag mound as well as the desire to further clarify the various species “-types” encountered (see below). In total, 11 probes from Site 34 (representing 554 examined samples) and eight probes and Layer I from Site 30 (representing 650 examined samples) were used. Charcoal fragments were hand-picked from the probed material. No preference for charcoal of a certain size was employed during collection or analysis so as to lessen the possibility of a bias in the results, though samples were not typically larger than 1–2 cm, while those smaller than 5 mm were avoided as they are frequently too small for a determination.

Taxonomic determinations were made on the basis of ligneous cell structure following^122–124^. Specimens were cut using razor blades and examined along three observational axes—transverse, tangential, and radial—under oblique angled top-lighting using a stereoscopic Carl Zeiss SteREO Discovery.V20 epi-illuminated microscope at magnifications of up to 360 × and a Vega3 TESCAN Scanning Electron Microscope at the Laboratory of Archaeobotany and Ancient Environments, TAU. Characteristic anatomical features, such as vessels and their arrangements, size and arrangement of rays, and abundance and nature of parenchyma were noted and identification at the highest possible systematic level was determined mainly by comparison to the wood and charcoal reference collection of the southern Levant (provided by the Steinhardt Museum of Natural History, Tel Aviv University, and collected from the region under study) as well as wood anatomy atlases^125–130^.

For certain taxa, due primarily to interfamilial or intergeneric similarities and overlaps in characteristic features, determinations were not always able to be definitively assigned at the species level; nonetheless, in these cases, “-types” were assigned in order to ascribe a significant affinity in the anatomical structure towards similarly observed features in modern reference samples and publications. Significantly, this includes the *Acacia* and *Pistacia* genera, for both of which three “-types” were encountered (*Acacia tortilis*-type, *A. raddianna*-type, and *A. pachyceras*-type; and *Pistacia atlantica*-type, *P. khinjuk*-type, and *P. palaestina*-type), as well as the members of the Chenopodiaceae/Amaranthaceae family. Accordingly, besides the information provided in the abovementioned references and modern comparative samples, a directed anatomical investigation of southern Levantine *Pistacia* species^58^ was referenced for the identification of this group. For the Chenopodiaceae/Amaranthaceae group, identification followed^125^ and modern reference samples, with further publications^56,57^ being referenced. Acacia species-types were assigned according to^125^ and modern reference samples.

For a complete enumeration of how species were generally assigned within this assemblage, please see the key in the Supplementary Information.

## Supplementary Information

Supplementary Information 4.

## Data Availability

All datasets and tables relevant to the research and the information provided in the manuscript are intended to be published within or alongside the manuscript as supplementary material.
