# Supplementary material for: Fuel exploitation and environmental degradation at the Iron Age copper industry of the Timna Valley, southern Israel
Source: Sci Rep. 2022 Sep 21;12:15434. doi: 10.1038/s41598-022-18940-z (PMC9492654; doi:10.1038/s41598-022-18940-z)
Supplement: Supplementary file 1 — Supplementary Information 4. [file 41598_2022_18940_MOESM1_ESM.docx]

**Fuel exploitation and environmental degradation at the Iron Age copper industry of the Timna Valley, southern Israel**

**Supplementary Information**

Mark Cavanagh^1, 2*^, Erez Ben-Yosef^1^, Dafna Langgut^1, 2^

1. Department of Archaeology and Ancient Near Eastern Cultures, Tel Aviv University, 6997801, Tel Aviv, Israel
2. Laboratory of Archaeobotany and Ancient Environments, Institute of Archaeology & The Steinhardt Museum of Natural History, Tel Aviv University, 6997801, Tel Aviv, Israel

*Corresponding author: Mark Cavanagh, [markgordonc@mail.tau.ac.il](mailto:markgordonc@mail.tau.ac.il)

Key to the identification of plant taxa identified within the Timna slag mound charcoal assemblages

1. Wood vessel-less, transition from early to latewood gradual, rays uniseriate, short (3-5 cells average height), cupressoid pits………...……………………………....***Juniperus***

▪Vessels present……………………………………………………………….... go to 2

1. Vascular bundles present, wood rayless, scalariform perforation plates and scalariform to opposite intervessel pits…………………………………………***Phoenix dactylifera***

▪Not as above…………………………………………….……………………….go to 3

1. Parenchyma and vessel elements storied…………………..……………………..go to 4

▪Parenchyma and vessel elements not storied…………….…………………….go to 19

1. Secondary phloem present………………………………….……………………go to 5

▪Secondary phloem absent………………………………….…………………..go to 14

1. Vessels mostly outside included phloem groups, arranged in radial bands of up to 10 or in clusters………………………………………...………….……….***Salvadora persica***

▪Not as above…………………………………………………………………….go to 6

1. AMAMARTHACEAE/CHENOPODIACEAE GROUP

▪Rays distinct………………………………………………...…………………..go to 7

▪Rays indistinct from radial phloem groups or absent….…..……………………go to 9

1. Rays short (1-5 tall) and 1-3 seriate, made of square an upright cells…………………. ………………………………………………….………………***Anabasis setifera*-type**

▪Not as above…………………………………………………………………….go to 8

1. Rays large (1-18 seriate, up to 70 cells high), vessels in clusters, up to 95 μm wide, intervessel pits ca. 6 μm wide……………………….………***Haloxylon persicum*-type**

▪Rays large (1-16 seriate, up to 1 mm tall), vessels up to 45 μm wide, intervessel pits 4-5 μm wide, gums sometimes present in vessels…………….***Aellenia lancifolia*-type**

1. Parenchyma in distinct conjunctive bands 4-5 cells wide, radial parenchyma creating the appearance of wide rays (>10 cells wide), vessels up to 110 μm………………….. ………………………………………………………….……….***Suaeda fruticosa*-type**

▪Not as above…………………………………………………………………...go to 10

1. Vessels larger than 50 μm (up to 110 μm), radial parenchyma rare but occasionally forming radial tracts up to 10 cells wide, composed of upright to weakly procumbent cells……………………………………………………….……..***Atriplex halimus*-type**

▪Not as above…………………………………………………………………...go to 11

1. Many vessels with gummy contents, radial strips of parenchyma up to 10 cells wide and over 1 mm tall, composed of upright cells, included phloem in distinct round, elliptic to crescent shaped groups…………………………..***Seidlitzia rosmarinus*-type**

▪Not as above…………………………………………………………………...go to 12

1. Included phloem of diffuse to concentric type, in round to crescent groups, radial strips almost absent, when present only 1-3 seriate and up to 5 cells high, spiral thickening present………………………………………………………......***Salsola tetrandra*-type**

▪Not as above…………………………………………………………………...go to 13

1. Included phloem of the concentric type, vessels round to angular, up to ca. 45 μm in tangential diameter, included phloem in distinct round to elliptic groups, sometimes coalescent, radial parenchyma 2-6 seriate, up to 30 cells high, composed of upright cells…………………………………………………………….***Salsola baryosma*-type**

▪Included phloem of the concentric type. vessels round, up to ca. 35 μm in tangential diameter, included phloem in distinct rounded groups, sometimes coalescent, radial strips of conjunctive parenchyma 2-6 seriate, up to 10 cells high, composed of upright, square, and irregularly shaped cells……………….................***Salsola vermiculata*-type**

1. Rays typically not wider than 1-3(4) seriate………………….………………...go to 15

▪Rays frequently 4 seriate or wider…………………………….……………….go to 18

1. Rays composed of procumbent cells…………………………………………...go to 16

▪Rays composed of square, upright, and procumbent cells mixed throughout ray………………………………………………………………………………go to 17

1. Growth rings distinct, vessels ring- to semi-ring porous, rounded to angular, at times larger than 200 μm, grouped in clusters and radial, tangential or oblique multiples of 2-6(13), rarely solitary. Rays larger than 1 mm, ray cells strongly procumbent……… …………………………………………………………………..***Calligonum comosum***

▪Growth rings distinct, vessels semi-ring-porous, rounded, mostly solitary (sometimes up to ca. 90%), or in tangential multiples of 2-6 and most rarely in clusters. Parenchyma scanty paratracheal and apotracheal diffuse or diffuse-in-aggregates, and marginal 1-2 seriate bands. Rays 1-3 seriate, up to 12 cells high, composed of procumbent cells…………………………………….……………….……..***Zygophyllum dumosum***

1. Growth rings distinct to indistinct, vessels diffuse- to semi-ring-porous, usually in clusters or variously directed multiples of 2-3. Paratracheal parenchyma scanty to vasicentric, confluent; apotracheal parenchyma seen in 1-3 seriate marginal bands. Vessels, parenchyma, and fibres storied. Rays (1)2-4 seriate……***Ochradenus baccatus***

▪Growth rings indistinct, diffuse porous vessels of two class sizes integrating together, the wider vessels either solitary or in multiples of 2-3(4), up to 100 μm, and narrower (10-30 μm) both solitary and in multiples of 2-4(6) and in clusters. Many vessels with gummy contents. Paratracheal parenchyma vasicentric, aliform to confluent in 2-5 celled bands. Vessels, parenchyma, and fibres exhibit a storied structure. Low rays tending to story………………………………………….………………***Nitraria retusa***

▪Growth rings faint to absent, vessels diffuse, rounded, of two class sizes: larger ones (up to 40-110 μm in diameter) either solitarily (ca. 30%) or in radial multiples or clusters of 2-5, and narrow vessels (20-40 μm) in clusters or multiples with wider vessels, gummy contents in many vessels, sclerotic tyloses sometimes present. Parenchyma vasicentric. Rays (1)2-4(5) seriate with procumbent to square central cells with 1-4 rows of weakly procumbent to upright marginal cells………………….. ……………………………………………………………………….***Capparis decidua***

1. Vessels diffuse porous vessels, arranged in oblique or dendritic patterns along with the paratracheal parenchyma; apotracheal parenchyma bands frequent, usually one-two cells wide, prominent helical thickenings within the vessels, rays (ca. 10-13/mm) at times 1-4 seriate though quite often larger. Rays composed of square to slightly procumbent central cells with one (or more) rows of square or upright marginal cells. Vessels up to ca. 100 μm wide, but frequently observed to be larger in roots…………. ………….………………………………………………………………***Retama raetam***

▪Vessels mostly solitary, sometimes clusters, up to 280 μm, parenchyma mostly vasicentric and storied together with the vessels, rays typically wide, sometimes compound, frequently up to 1 mm or more in height……………...………***Tamarix* sp.**

1. Parenchyma in large, continuous bands of 5-8 or more….……………………..go to 20

▪Not as above.. …………………………………………………………………go to 21

1. ***Acacia* spp.** Vessels infrequent (~5-20/mm2 ), diffuse-porous, either solitary, in small clusters, or in radial multiples of 2-4. Paratrachial parenchyma is lozenge-aliform, confluent, often present in large continuous tangential bands able to achieve a width of 30 cells or greater. 1-6 seriate rays with strongly procumbent ray cells.

▪ca. 75% solitary vessels………………………………….……………..***A. tortilis*-type**

▪ca. 40% solitary vessels, spiral thickenings………………………..***A. raddianna*-type**

▪ca. 40% solitary vessels, gummy contents……………….………..***A. pachyceras*-type**

1. Rays mostly uniseriate, rarely biseriate or larger………….……………………go to 22

▪Rays frequently 2 or more seriate…………………………..…………………..go to 23

1. Growth rings distinct, vessels diffuse, rounded, solitary (ca. 25-30%) and in radial multiples of 2-3, occasionally in small clusters. Many with gummy contents. Paratracheal parenchyma scanty to vasicentric, apotracheal parenchyma in 1(3) seriate marginal bands. Rays uni- and more rarely bi-seriate, up to 20(24) cells high, and composed of square, upright and weakly procumbent cells…….***Ziziphus spina-christi***

▪Growth rings faint, vessels round, ca. 20% solitary and in mostly radial multiples of 2-5(10), less frequently in clusters, tyloses sometimes present. Intervessel pits are polygonal to rounded, with slit-like and sometimes coalescent apertures. Fibres medium-thick walled. Parenchyma scanty paratracheal. Rays mostly uniseriate but up to 2(4) seriate and up to 25 cells high, composed of upright marginal cells and procumbent to upright central cells. Sheath cells sometimes present…..***Rhus tripartita***

1. Growth rings distinct, vessels ring- to semi-ring-porous…………….…………go to 24

▪Growth rings faint…………………………………………………….………..go to 25

1. Vessels in multiples and clusters, some of which – especially the widest – are solitary. All have prominent spiral thickenings in the vessels and vascular tracheids. Rays are uni- and multi-seriate, composed of procumbent central cells and square, upright, and few slightly procumbent marginal cells. Resin canals are present in multiseriate portions……………………………………………………………………***Pistacia* spp.**

▪Vessels ring- to semi-ring porous, one or more rows of very large vessels at beginning of ring, in latewood arranged mostly in radial multiples, rays up to 6 cells wide, crystals in most marginal cells….……………………………………………...***P. atlantica*-type**

▪Ring- to semi-ring-porous, gradual decrease in vessel size, rays typically between 1-4 seriate, marginal ray cells of irregular shape, many with crystals, rays do not exceed 35 cells in height….……………………………………………….……***P. palaestina*-type**

▪One row of large vessels, the remainder small, vessels arranged in oblique/dendritic pattern, rays mostly 1-3 seriate, marginal ray cells square or rectangular, fewer crystals…………………………………………………………………***P. khinjuk*-type**

1. Growth rings faint to absent, vessels diffuse, rounded, of two class sizes: larger ones (40-100 μm in diameter) rarely solitarily, frequently in radial multiples or clusters, and narrow vessels (20-40 μm) in clusters or multiples of up to 12 with wider vessels. Rays (1)2-4(5) seriate with procumbent to square central cells with 1-4 rows of weakly procumbent to upright marginal cells…………………………....***Capparis cartaliginea***

▪Growth rings faint to absent (occasionally distinct), vessels rounded to angular, diffuse to semi-ring-porous, mainly in radial multiples of up to 7 or clusters, or rarely solitary, parenchyma are scanty paratracheal and diffuse apotracheal, fibres very thin walled. Rays 1- 4(6) seriate, up to 25 cells high, composed of procumbent central cells and varying numbers (5-10) of square and upright marginal cells……………….. ….…………………………………………………………………..***Calotropis procera***

SI Fig.1 – Results of identified charcoal according to probed contexts.


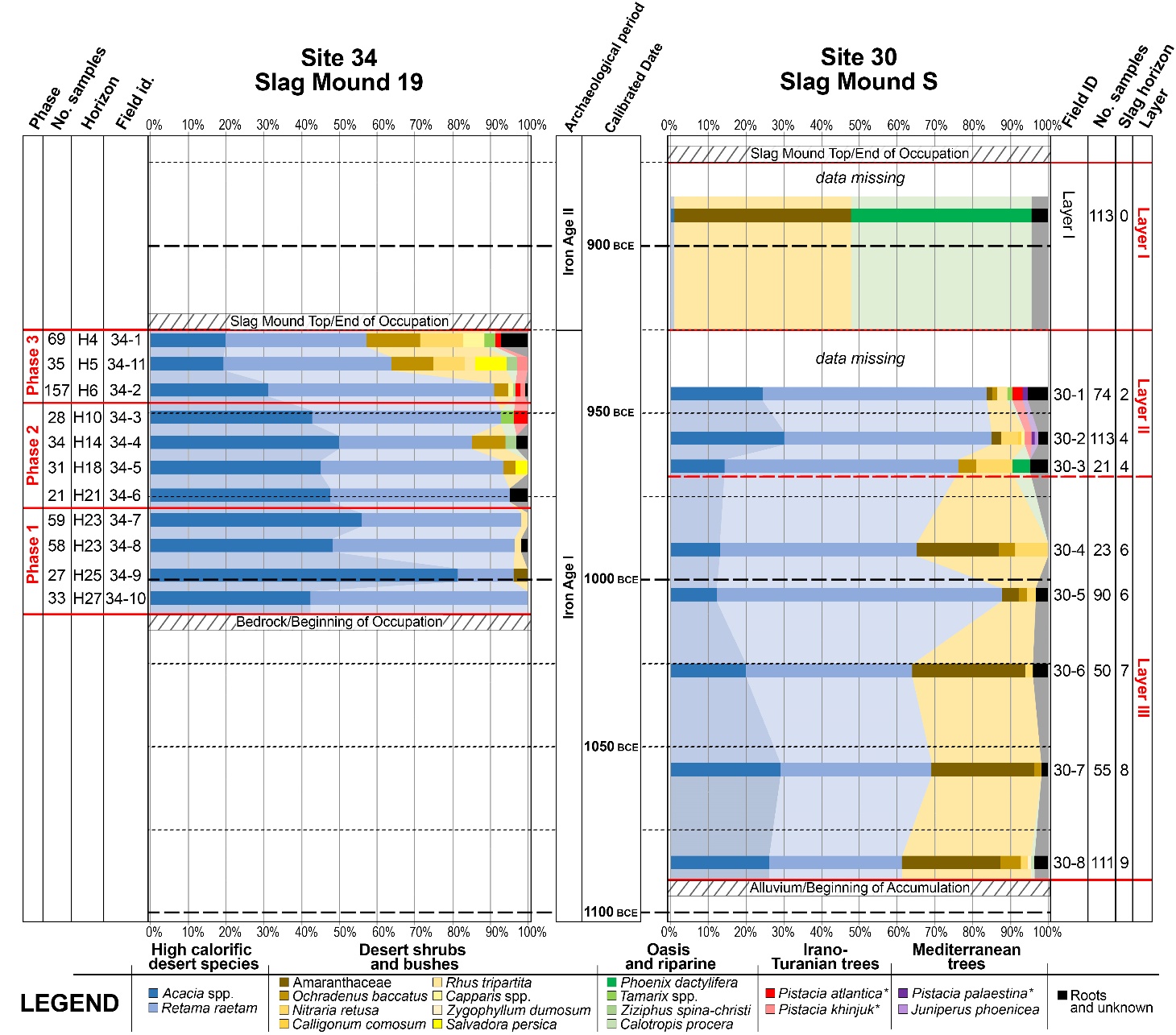


**Fig. S1**: Charcoal spectra reflected within Site 34 (a) and Site 30 (b) according to probed contexts. Results are given according to percentage values within each sampled probe and arranged according to sampled horizon and modeled radiometric dates. Field number for each probe, absolute value of examined samples, and respective horizon/slag layer are given (see fig. 3 for slag mound horizons and sampling locations). Legend entries with asterisks indicate species-types.

Fig. S2 – Scanning Electron Microscopic images of select plant taxa


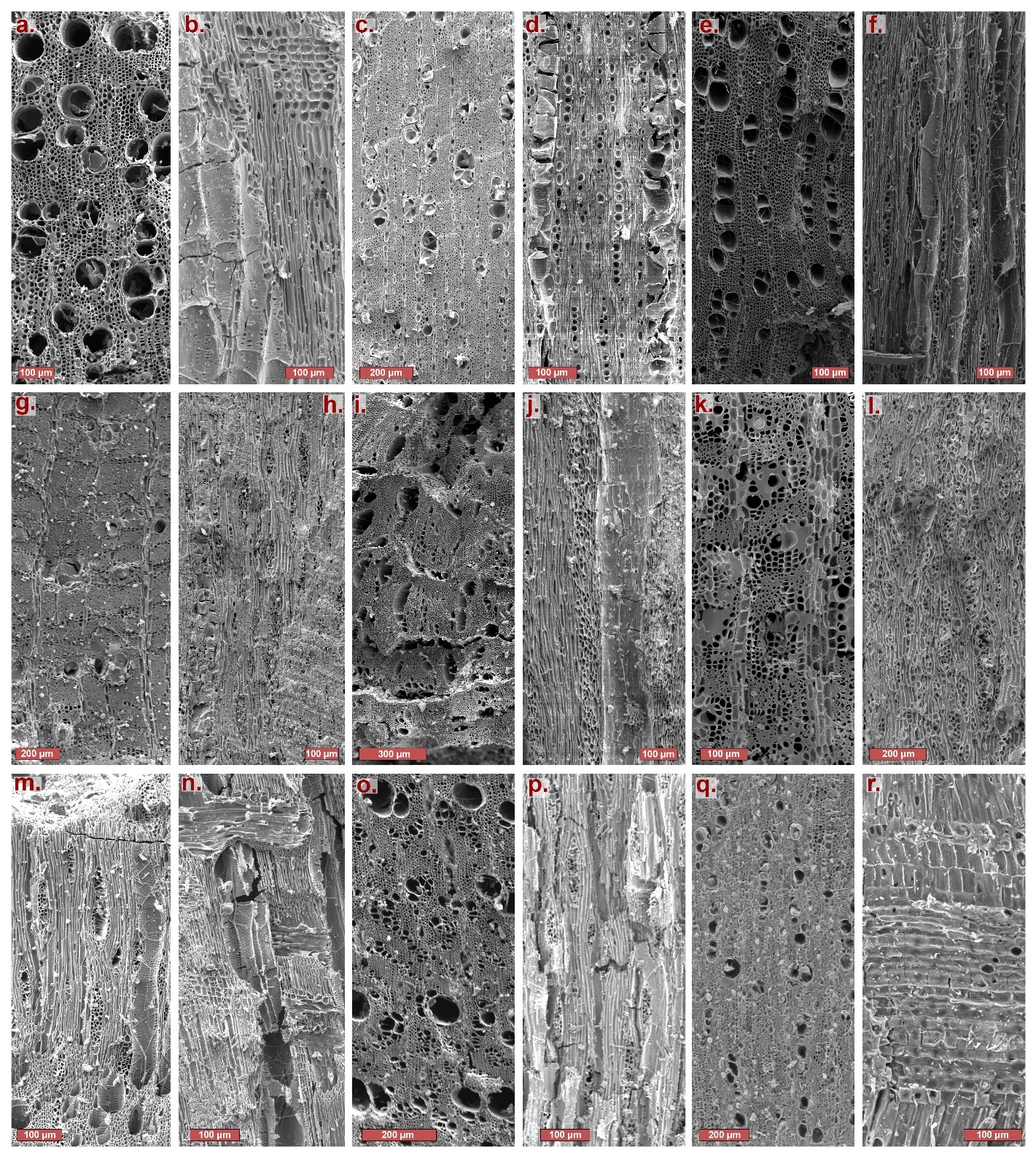


**Fig. S2**: Scanning electron microscope images of select identified taxa and taxa-types with collection locations indicated in parentheses: a) Ochradenus baccatus (Site 34, Probe 11), transverse; scale 100 μm; b) Ochradenus baccatus (Site 34, Probe 11), radial; scale 100 μm; c) Ziziphus spina-christi (Site 32, Probe 2), transverse, scale 200 μm; d) Ziziphus spina-christi (Site 32, Probe 2), tangential, scale 100 μm; e) Rhus tripartita (Site 34, Probe 2), transverse, scale 100 μm; f) Rhus tripartita (Site 34, Probe 2), tangential, scale 100 μm, note as well presence of fungal hyphae in vessels; g) Nitraria retusa (Site 34, Probe 11), transverse, scale 200 μm; h) Nitraria retusa (Site 34, Probe 11), tangential, scale 100 μm; i) Aellenia lancifolia-type (Amaranthaceae; Site 30, Probe 8), transverse, scale 300 μm; j) Aellenia lancifolia-type (Amaranthaceae; Site 30, Probe 8), tangential, scale 100 μm; k) Salvadora persica (Site 34, Probe 4), transverse, scale 100 μm; l) Salvadora persica (Site 34, Probe 4), tangential, scale 200 μm; m) Pistacia palaestina-type (Site 34, Probe 2), tangential, scale 100 μm, note as well fungal hyphae in vessel; n) Pistacia palaestina-type (Site 34, Probe 2), radial, scale 100 μm, note irregular-shaped marginal ray cells; o) Pistacia khinjuk-type (Site 34, Probe 2), transverse, scale 200 μm; p) Pistacia khinjuk-type (Site 34, Probe 2), tangential, scale 100 μm; q) Capparis cartaliginea (Site 30, Probe 2), transverse, scale 200 μm; r) Capparis cartaliginea (Site 30, Probe 2), radial, scale 100 μm.

Fig. S3 – Optical microscope images of select plant taxa


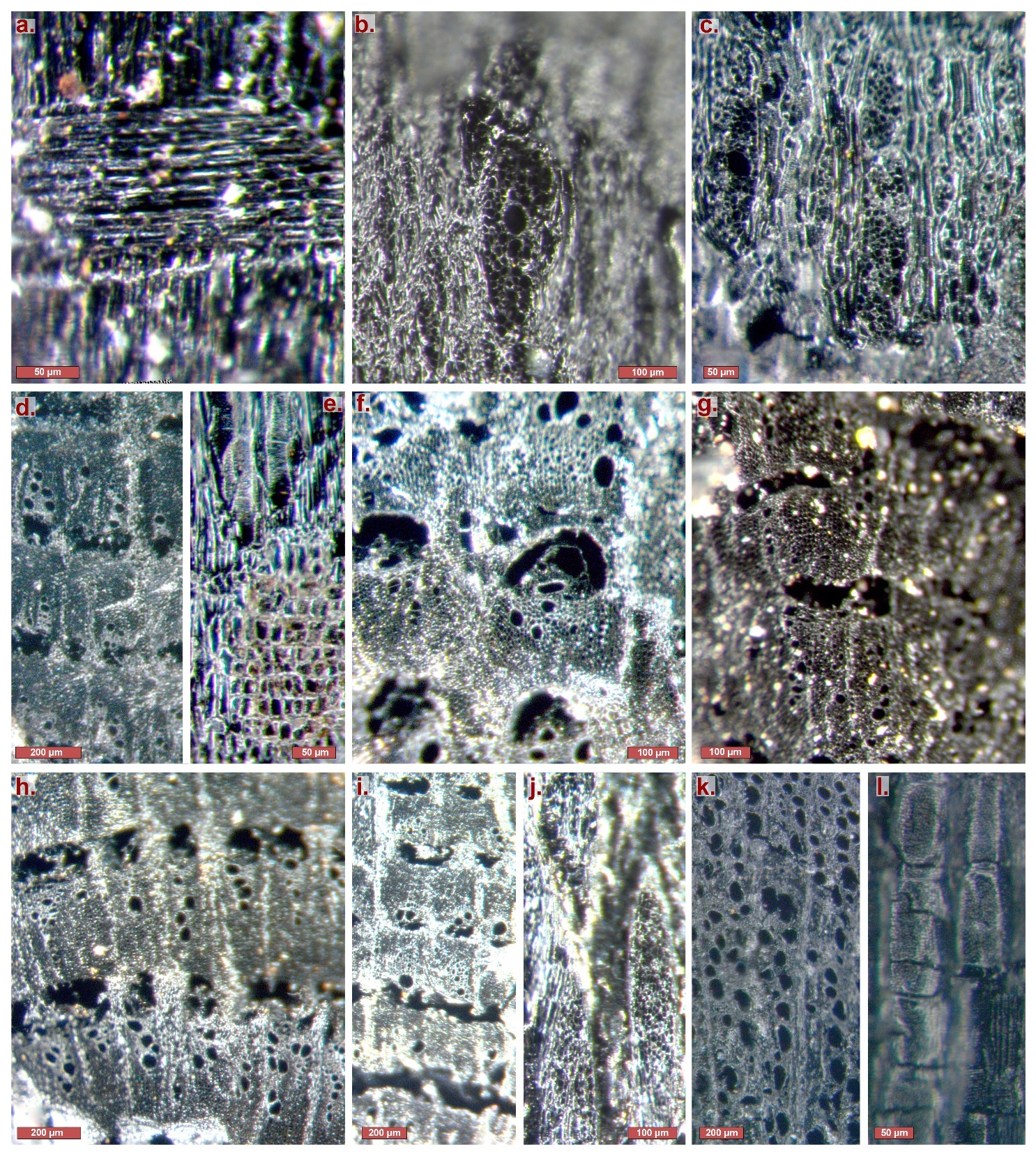


**Fig. S3:** Optical microscope images of select identified taxa and taxa-types with collection locations indicated in parentheses: Scanning electron microscope images of select identified taxa and taxa-types with collection locations indicated in parentheses: a) Pistacia palaestina-type (Site 30, Probe 2), radial; scale 50 μm, not irregular-shaped marginal ray cells and crystals in marginal ray cells; b) Pistacia atlantica-type (Site 30, Probe 1), tangential; scale 100 μm; c) Retama raetam (Site 30, Probe 7), tangential, scale 50 μm; d) Salsola vermiculata-type (Amaranthaceae; Site 30, Layer I), transverse, scale 200 μm; e) Salsola vermiculata-type (Amaranthaceae; Site 30, Layer I), radial, scale 50 μm; f) Seidlitzia rosmarinus-type (Amaranthaceae; Site 30, Layer I), transverse, scale 100 μm; g) Anabasis setifera-type (Amaranthaceae; Site 30, Probe 4), transverse, scale 100 μm; h) Salsola baryosma-type (Amaranthaceae; Site 30, Layer I), transverse, scale 200 μm; i) Haloxylon persicum-type (Amaranthaceae; Site 30, Layer I), transverse, scale 200 μm; j) Haloxylon persicum-type (Amaranthaceae; Site 30, Layer I), tangential, scale 100 μm; k) Tamarix sp. (Site 34, Probe 3), transverse, scale 200 μm; l) Tamarix sp. (Site 34, Probe 3), radial, scale 50 μm.

Table S1 – Radiocarbon dates for Timna Site 30, Slag Mound S.

Radiometric results along with calibrated and modeled dates for dated samples collected from Slag Mound S of Timna Site 30 (taken from ^[1: Table 4]^). Dates calibrated and modeled using OxCal 4.1.6^[2,3]^.

**Table S1**: Radiocarbon Dates from Site 30, Area S Slag Mound (from ^[12: 55, Table 4]^)

| **Sample Name** | **Material** | **Age B.P.** | **1σ cal.**  **B.C.E.** | **2σ cal.**  **B.C.E.** | **1σ**  **Modeled** | **2σ**  **Modeled** | **Stratigraphic**  **Height** | **Layer /**  **Context** |
| --- | --- | --- | --- | --- | --- | --- | --- | --- |
| IS26-S2-W1  AA86520 | Wood twig | 2705±35 | 895–816 | 915–804 | 912–836 | 970–808 | 152 | Layer I  (bottom) |
| IS26-S2-g1-h  AA86519 | Olive pit | 2814±34 | 1006–921 | 1070–847 | 971–910 | 1006–894 | 135 | Layer II |
| IS26-S1-g1-b  AA86518 | Grape seed(?) | 2819±35 | 1011–921 | 1113–896 | 1009–946 | 1037–920 | 71 | Layer II |
| IS26-S1-d3a  AA86517 | Date seed | 2893±39 | 1129–1008 | 1252–941 | 1057–981 | 1101–938 | 57 | Layer III(?) |
| IS26-S1-w7  AA86516 | Wood bark | 2859 ± 34 | 1111–946 | 1129–919 | 1108–1009 | 1130–943 | 28 | Layer III |

Table S2 – Radiocarbon dates for Timna Site 34 (“Slaves Hill”), Slag Mound 19.

Radiometric results along with calibrated and modeled dates for dated samples collected from Slag Mound 19 of Timna Site 34 (taken from ^[4: 183, Table 1]^). Calibrated and modeled by OxCal v.4.2 and Calibration Curve IntCal13. © C. Bronk Ramsey 2013^[5]^. Note OxA-27962 and OxA-27963 were collected from *in situ* excavated contexts near the top of the slag mound, and not from along the section.

**Table S2**: Radiocarbon Dates from Site 34, Area 19 Slag Mound (from ^[13: 183, Table 1]^)

| **Lab #** | **Horizon/**  **Locus** | **Material** | **Δ13C** | **Age B.P.** | **1σ cal.**  **B.C.E.** | **2σ cal.**  **B.C.E.** | **1σ**  **Modeled** | **2σ**  **Modeled** |
| --- | --- | --- | --- | --- | --- | --- | --- | --- |
| OxA-27964 | H29 | Date seed | -26.16 | 2847*±*27 | 1048–942 | 1108–925 | 1036–996 | 1056–948 |
| OxA-27965 | H27 | Date seed | -24.42 | 2876*±*25 | 1107–1010 | 1126 –942 | 1026–992 | 1047–949 |
| OxA-27987 | H23 | Date seed | -24.21 | 2814*±*28 | 1001 –929 | 1045 –902 | 1015–987 | 1033–946 |
| OxA-27989 | H21 | Date seed | -23.99 | 2816*±*26 | 1002 –931 | 1042 –905 | 1010–982 | 1020–948 |
| OxA-27924 | H13 | Olive pit | -19.07 | 2855*±*35 | 1074 –940 | 1122 –919 | 1005–975 | 1012–944 |
| OxA-28010 | H13 | Date seed | -24.55 | 2846*±*27 | 1047 –942 | 1108 –924 | 1005–975 | 1012–943 |
| OxA-27988 | H12 | Date seed | -22.78 | 2831*±*28 | 1016 –931 | 1072 –907 | 1003–967 | 1007–936 |
| OxA-27963 | L112 | Date seed | -22.32 | 2818*±*27 | 1004 –930 | 1045 –906 | 1000–939 | 1005–927 |
| OxA-27962 | L106 | Date seed | -22.93 | 2800*±*24 | 980 –916 | 1016 –896 | 997–932 | 1003–917 |

**Table S3**: Total charcoal spectra per Probe and Layer from Site 30, Slag Mound S

| **Probe** | **Ac** | **At** | **Ar** | **Ap** | **Rr** | **Ob** | **Nr** | **Cl** | **Cc** | **Zd** | **Rt** | **AM** | **Sb** | **Sv** | **St** | **Ah** | **Al** | **Sf** | **Sr** | **Hp** | **As** | **AU** | **Pd** | **Ta** | **Cp** | **Pa** | **Pp** | **Pk** | **Jp** | **Uk** | **Total** |
| --- | --- | --- | --- | --- | --- | --- | --- | --- | --- | --- | --- | --- | --- | --- | --- | --- | --- | --- | --- | --- | --- | --- | --- | --- | --- | --- | --- | --- | --- | --- | --- |
| Layer I | 1 | 0 | 0 | 0 | 0 | 0 | 0 | 0 | 0 | 0 | 0 | 53 | 10 | 19 | 5 | 8 | 1 | 5 | 3 | 1 | 0 | 1 | 54 | 0 | 0 | 0 | 0 | 0 | 0 | 5 | 113 |
| 30-1 | 18 | 6 | 5 | 5 | 44 | 1 | 0 | 0 | 0 | 0 | 2 | 1 | 0 | 0 | 0 | 0 | 0 | 1 | 0 | 0 | 0 | 0 | 0 | 1 | 0 | 2 | 1 | 0 | 0 | 4 | 74 |
| 30-2 | 34 | 6 | 1 | 1 | 62 | 0 | 5 | 1 | 1 | 0 | 0 | 3 | 0 | 1 | 1 | 1 | 0 | 0 | 0 | 0 | 0 | 0 | 0 | 0 | 0 | 0 | 1 | 2 | 1 | 3 | 113 |
| 30-3 | 3 | 0 | 0 | 0 | 13 | 1 | 2 | 0 | 0 | 0 | 0 | 0 | 0 | 0 | 0 | 0 | 0 | 0 | 0 | 0 | 0 | 0 | 1 | 0 | 0 | 0 | 0 | 0 | 0 | 1 | 21 |
| 30-4 | 3 | 1 | 1 |  | 12 | 1 | 2 | 0 | 0 | 0 | 0 | 5 | 3 | 1 | 0 | 0 | 0 | 0 | 0 | 0 | 1 | 0 | 0 | 0 | 0 | 0 | 0 | 0 | 0 | 0 | 23 |
| 30-5 | 11 | 0 | 1 | 1 | 68 | 2 | 2 | 0 | 0 | 0 | 0 | 4 | 4 | 0 | 0 | 0 | 0 | 0 | 0 | 0 | 0 | 0 | 0 | 0 | 0 | 0 | 0 | 0 | 0 | 3 | 90 |
| 30-6 | 10 | 0 | 0 | 0 | 22 | 0 | 1 | 0 | 0 | 0 | 0 | 15 | 6 | 3 | 0 | 1 | 0 | 1 | 0 | 3 | 0 | 1 | 0 | 0 | 0 | 0 | 0 | 0 | 0 | 2 | 50 |
| 30-7 | 16 | 1 | 0 | 0 | 22 | 1 | 0 | 0 | 0 | 0 | 0 | 15 | 10 | 0 | 0 | 3 | 0 | 0 | 0 | 2 | 0 | 0 | 0 | 0 | 0 | 0 | 0 | 0 | 0 | 1 | 55 |
| 30-8 | 29 | 15 | 10 | 1 | 39 | 6 | 2 | 0 | 0 | 1 | 0 | 29 | 7 | 2 | 0 | 11 | 5 | 3 | 1 | 0 | 0 | 0 | 0 | 0 | 1 | 0 | 0 | 0 | 0 | 4 | 111 |
| **%** | 125 | 29 | 18 | 8 | 282 | 12 | 14 | 1 | 1 | 1 | 2 | 125 | 40 | 26 | 6 | 24 | 6 | 10 | 4 | 6 | 1 | 2 | 55 | 1 | 1 | 2 | 2 | 2 | 1 | 23 | 650 |
| **Percent** | 19.23 | 4.46 | 2.77 | 1.23 | 43.38 | 1.85 | 2.15 | 0.15 | 0.15 | 0.15 | 0.31 | 19.23 | 6.15 | 4.00 | 0.92 | 3.69 | 0.92 | 1.54 | 0.62 | 0.92 | 0.15 | 0.31 | 8.46 | 0.15 | 0.15 | 0.31 | 0.31 | 0.31 | 0.15 | 3.54 | 100 |
|  |  |  |  |  |  |  |  |  |  |  |  |  |  |  |  |  |  |  |  |  |  |  |  |  |  |  |  |  |  |  |  |
| **Total Layer I** | 1 | 0 | 0 | 0 | 0 | 0 | 0 | 0 | 0 | 0 | 0 | 53 | 10 | 19 | 5 | 8 | 1 | 5 | 3 | 1 | 0 | 1 | 54 | 0 | 0 | 0 | 0 | 0 | 0 | 5 | 113 |
| **% Layer I** | 0.88 | 0 | 0 | 0 | 0 | 0 | 0 | 0 | 0 | 0 | 0 | 46.9 | 8.85 | 16.81 | 4.42 | 7.08 | 0.88 | 4.42 | 2.65 | 0.88 | 0 | 0.88 | 47.79 | 0 | 0 | 0 | 0 | 0 | 0 | 4.42 | 100 |
| **Total Layer II** | 55 | 12 | 6 | 6 | 119 | 2 | 7 | 1 | 1 | 0 | 2 | 4 | 0 | 1 | 1 | 1 | 0 | 1 | 0 | 0 | 0 | 0 | 1 | 1 | 0 | 2 | 2 | 2 | 1 | 8 | 208 |
| **% Layer II** | 26.44 | 5.77 | 2.88 | 2.88 | 57.21 | 0.96 | 3.37 | 0.48 | 0.48 | 0 | 0.96 | 1.92 | 0 | 0.48 | 0.48 | 0.48 | 0 | 0.48 | 0 | 0 | 0 | 0 | 0.48 | 0.48 | 0 | 0.96 | 0.96 | 0.96 | 0.48 | 3.85 | 100 |
| **Total Layer III** | 69 | 17 | 12 | 2 | 163 | 10 | 7 | 0 | 0 | 1 | 0 | 68 | 30 | 6 | 0 | 15 | 5 | 4 | 1 | 5 | 1 | 1 | 0 | 0 | 1 | 0 | 0 | 0 | 0 | 10 | 329 |
| **% Layer III** | 20.97 | 5.17 | 3.65 | 0.61 | 49.54 | 3.04 | 2.13 | 0 | 0 | 0.3 | 0 | 20.67 | 9.12 | 1.82 | 0 | 4.56 | 1.52 | 1.22 | 0.3 | 1.52 | 0.3 | 0.3 | 0 | 0 | 0.3 | 0 | 0 | 0 | 0 | 3.04 | 100 |

Table S3 – Site 30 total charcoal assemblage: Absolute number of identified charcoal samples per species/taxa from Slag Mound S of Site 30, as well as percentages of each taxa per Layer and total Site 30 assemblage. Note: species-types of *Acacia* and Amaranthaceae are included in total species count for these taxa. Ac=*Acacia* spp. (total); At=*Acacia tortilis*-type; Ar=*Acacia raddiana*-type; Ap=*Acacia pachyceras*-type; Rr=*Retama raetam*; Ob=*Ochradenus baccatus*; Nr=*Nitraria retusa*; Cl-*Calligonum comosum*; Cc=*Capparis cartaliginea*; Zd=*Zygophyllum dumosum*; Rt=*Rhus tripartita*; AM=Amaranthaceae/Chenopodiaceae (total); Sb=*Salsola baryosma*-type; Sv=*Salsola vermiculata*-type; St=*Salsola tetrandra*-type; Ah=*Atriplex halimus*-type; Al=*Aellenia lancifolia*-type; Sf=*Suaeda fruticosa*-type; *Seidlitzia rosmarinus*-type; Hp=*Haloxylon persicum*-type; As=*Anabasia setifera*-type; AU=Amaranthaceae (unknown type); Pd=*Phoenix dactylifera*; Ta=*Tamarix aphylla*; Cp=*Calotropis procera*; Pa=*Pistacia atlantica*-type; Pp=*Pistacia palaestina*-type; Pk=*Pistacia khinjuk*-type; Jp=*Juniperus phoenicea*, Uk=unknown.

Table S4: Total charcoal spectra per Probe and Phase from Site 34, Slag Mound 19

| **Probe** | **Phase** | **Ac** | **Rr** | **AM** | **Ob** | **Nr** | **Cd** | **Rt** | **Sp** | **Tx** | **Zs** | **Pa** | **Pk** | **Uk** | **Total** |
| --- | --- | --- | --- | --- | --- | --- | --- | --- | --- | --- | --- | --- | --- | --- | --- |
| **C1** | 3 | 14 | 26 | 0 | 10 | 8 | 4 | 0 | 0 | 2 | 0 | 1 | 0 | 5 | 70 |
| **C11** | 3 | 7 | 16 | 0 | 4 | 3 | 0 | 1 | 3 | 0 | 1 | 0 | 1 | 0 | 36 |
| **C2** | 3 | 49 | 94 | 0 | 6 | 0 | 2 | 0 | 0 | 0 | 1 | 2 | 2 | 1 | 157 |
| **C3** | 2 | 12 | 14 | 0 | 0 | 0 | 0 | 0 | 0 | 1 | 0 | 1 | 0 | 0 | 28 |
| **C4** | 2 | 17 | 12 | 0 | 3 | 0 | 0 | 0 | 0 | 0 | 1 | 0 | 0 | 1 | 34 |
| **C5** | 2 | 14 | 15 | 0 | 1 | 0 | 0 | 0 | 1 | 0 | 0 | 0 | 0 | 0 | 31 |
| **C6** | 2 | 10 | 10 | 0 | 0 | 0 | 0 | 0 | 0 | 0 | 0 | 0 | 0 | 1 | 21 |
| **C7** | 1 | 33 | 25 | 0 | 0 | 0 | 0 | 1 | 0 | 0 | 0 | 0 | 0 | 0 | 59 |
| **C8** | 1 | 28 | 28 | 0 | 0 | 0 | 0 | 1 | 0 | 0 | 0 | 0 | 0 | 1 | 58 |
| **C9** | 1 | 22 | 4 | 1 | 0 | 0 | 0 | 0 | 0 | 0 | 0 | 0 | 0 | 0 | 27 |
| **C10** | 1 | 14 | 19 | 0 | 0 | 0 | 0 | 0 | 0 | 0 | 0 | 0 | 0 | 0 | 33 |
|  | **Total Phase 3** | 70 | 136 | 0 | 20 | 11 | 6 | 1 | 3 | 2 | 2 | 3 | 3 | 6 | 263 |
|  | **% Phase 3** | 28.69 | 55.74 | 0 | 8.20 | 4.51 | 2.46 | 0.41 | 1.23 | 0.82 | 0.82 | 1.23 | 1.23 | 2.46 | 100 |
|  | **Total Phase 2** | 53 | 51 | 0 | 4 | 0 | 0 | 0 | 1 | 1 | 1 | 1 | 0 | 2 | 114 |
|  | **% Phase 2** | 46.49 | 44.74 | 0.00 | 3.51 | 0 | 0 | 0 | 0.88 | 0.88 | 0.88 | 0.88 | 0 | 1.75 | 100 |
|  | **Total Phase 1** | 97 | 76 | 1 | 0 | 0 | 0 | 2 | 0 | 0 | 0 | 0 | 0 | 1 | 177 |
|  | **% Phase 2** | 54.80 | 42.94 | 0.56 | 0.00 | 0.00 | 0.00 | 1.13 | 0 | 0 | 0 | 0 | 0 | 0.56 | 100 |
|  | **Total (all phases)** | 220 | 263 | 1 | 24 | 11 | 6 | 3 | 4 | 3 | 3 | 4 | 3 | 9 | 554 |
|  | **% (total)** | 39.71 | 47.47 | 0.18 | 4.33 | 1.99 | 1.08 | 0.54 | 0.72 | 0.54 | 0.54 | 0.72 | 0.54 | 1.62 | 100 |

Table S4 – Site 34 total charcoal assemblage: Absolute number of identified charcoal samples per species/taxa from Slag Mound 19 of Site 34, as well as percentages of each taxa per Phase and total Site 34 assemblage. Ac=*Acacia* spp. (total); Rr=*Retama raetam*; AM=Amaranthaceae/Chenopodiaceae (total); Ob=*Ochradenus baccatus*; Nr=*Nitraria retusa*; Cd=*Capparis decidua*; Rt=*Rhus tripartita*; Sp=*Salvadora persica*; Tx=*Tamarix* sp.; Zs=*Ziziphus spina-christi*; Pa=*Pistacia atlantica*-type; Pk=*Pistacia khinjuk*-type; Uk=unknown.

**Table S5**: Combined industrial charcoal assemblages of Sites 30 and 34

|  | **Ac** | **Rr** | **Ob** | **Nr** | **AM** | **Cs** | **Rt** | **Zd** | **Cl** | **Sp** | **Pd** | **Zs** | **Tx** | **Cp** | **Pa** | **Pp** | **Pk** | **Jp** | **Uk** | **Total** |
| --- | --- | --- | --- | --- | --- | --- | --- | --- | --- | --- | --- | --- | --- | --- | --- | --- | --- | --- | --- | --- |
| **Total Site 30** | 125 | 282 | 12 | 14 | 125 | 1 | 2 | 1 | 1 | 0 | 55 | 0 | 1 | 1 | 2 | 2 | 2 | 1 | 23 | 650 |
| **% Site 30** | 19.23 | 43.38 | 1.85 | 2.15 | 19.23 | 0.15 | 0.31 | 0.15 | 0.15 | 0 | 8.46 | 0 | 0.15 | 0.15 | 0.31 | 0.31 | 0.31 | 0.15 | 3.54 | 100 |
| **Total Site 34** | 220 | 263 | 24 | 11 | 1 | 6 | 3 | 0 | 0 | 4 | 0 | 3 | 3 | 0 | 4 | 0 | 3 | 0 | 9 | 554 |
| **% Site 34** | 39.71 | 47.47 | 4.33 | 1.99 | 0.18 | 1.08 | 0.54 | 0 | 0 | 0.72 | 0 | 0.54 | 0.54 | 0 | 0.72 | 0 | 0.54 | 0 | 1.62 | 100 |
| **Total assemblage** | 345 | 545 | 36 | 25 | 126 | 7 | 5 | 1 | 1 | 4 | 55 | 3 | 4 | 1 | 6 | 2 | 5 | 1 | 32 | 1204 |
| **% total assemblage** | 28.65 | 45.27 | 2.99 | 2.08 | 10.47 | 0.58 | 0.42 | 0.08 | 0.08 | 0.33 | 4.57 | 0.25 | 0.33 | 0.08 | 0.5 | 0.17 | 0.42 | 0.08 | 2.66 | 100 |

Table S5 – Total combined industrial Iron Age charcoal assemblages of Timna Sites 30 and 34: Ac=*Acacia* spp. (total); Rr=*Retama raetam*; Ob=*Ochradenus baccatus*; Nr=*Nitraria retusa*; AM=Amaranthaceae/Chenopodiaceae (total); Cs=*Capparis* spp. (total); Rt=*Rhus tripartita*; Zd=*Zygophyllum dumosum*; Cl-*Calligonum comosum*; Sp=*Salvadora persica*; Pd=*Phoenix dactylifera*; Zs=*Ziziphus spina-christi*; Tx=*Tamarix* spp. (all); Cp=*Calotropis procera*; Pa=*Pistacia atlantica*-type; Pp=*Pistacia palaestina*-type; Pk=*Pistacia khinjuk*-type; Jp=*Juniperus phoenicea*, Uk=unknown.

References:

1. Ben-Yosef, E., Shaar, R., Tauxe, L. & Ron, H. A new chronological framework for Iron Age copper production at Timna (Israel). *B. Am. Sch. Oriental Re.* **367**, 31-71 (2012).
2. Bronk Ramsey, C. Bayesian analysis of radiocarbon dates. *Radiocarbon* **51**(1), 337–360 (2009).
3. Reimer, P. J. *et al.* Intcal09 and Marine09 radiocarbon age calibration curves, 0–50,000 Years cal bp. *Radiocarbon* **51**(4), 1111-1150 (2009).
4. Ben-Yosef, E. Back to Solomon’s era: Results of the first excavations at ‘Slaves’ Hill’ (Site 34, Timna, Israel). *B. Am. Sch. Oriental Re.* **376**(1), 169-198 (2016).
5. Bronk Ramsey, C., Scott, E.M. & van der Plicht, J. Calibration for archaeological and environmental terrestrial samples in the time range 26–50 ka cal BP. *Radiocarbon* **55**(4), 2021-2027 (2013).
